# Evolution of five environmentally responsive gene families in a pine‐feeding sawfly, *Neodiprion lecontei* (Hymenoptera: Diprionidae)

**DOI:** 10.1002/ece3.10506

**Published:** 2023-10-01

**Authors:** Kim L. Vertacnik, Danielle K. Herrig, R. Keating Godfrey, Tom Hill, Scott M. Geib, Robert L. Unckless, David R. Nelson, Catherine R. Linnen

**Affiliations:** ^1^ Department of Entomology University of Kentucky Lexington Kentucky USA; ^2^ Department of Biology University of Kentucky Lexington Kentucky USA; ^3^ McGuire Center for Lepidoptera and Biodiversity, University of Florida Gainesville Florida USA; ^4^ National Institute of Allergy and Infectious Diseases Bethesda Maryland USA; ^5^ Tropical Crop and Commodity Protection Research Unit United States Department of Agriculture: Agriculture Research Service Pacific Basin Agricultural Research Center Hilo Hawaii USA; ^6^ Department of Molecular Biosciences University of Kansas Lawrence Kansas USA; ^7^ Department of Microbiology, Immunology and Biochemistry University of Tennessee Health Science Center Memphis Tennessee USA

**Keywords:** chemosensation, detoxification, evolutionary predictability, gene family evolution, Hymenoptera, immunity

## Abstract

A central goal in evolutionary biology is to determine the predictability of adaptive genetic changes. Despite many documented cases of convergent evolution at individual loci, little is known about the repeatability of gene family expansions and contractions. To address this void, we examined gene family evolution in the redheaded pine sawfly *Neodiprion lecontei*, a noneusocial hymenopteran and exemplar of a pine‐specialized lineage evolved from angiosperm‐feeding ancestors. After assembling and annotating a draft genome, we manually annotated multiple gene families with chemosensory, detoxification, or immunity functions before characterizing their genomic distributions and molecular evolution. We find evidence of recent expansions of bitter gustatory receptor, clan 3 cytochrome P450, olfactory receptor, and antimicrobial peptide subfamilies, with strong evidence of positive selection among paralogs in a clade of gustatory receptors possibly involved in the detection of bitter compounds. In contrast, these gene families had little evidence of recent contraction via pseudogenization. Overall, our results are consistent with the hypothesis that in response to novel selection pressures, gene families that mediate ecological interactions may expand and contract predictably. Testing this hypothesis will require the comparative analysis of high‐quality annotation data from phylogenetically and ecologically diverse insect species and functionally diverse gene families. To this end, increasing sampling in under‐sampled hymenopteran lineages and environmentally responsive gene families and standardizing manual annotation methods should be prioritized.

## Graphical abstract

We examine the predictability of gene family size changes in the pine tree specialist *Neodiprion lecontei*. Overall, our results are consistent with predictable gene family expansions and contractions in response to novel selection pressures.

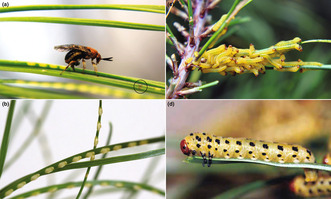

## 1 INTRODUCTION

Multigene families are a potentially important source of evolutionary innovation. When gene families grow via duplication, for example, reduced functional constraints may facilitate the development of phenotypic novelty (Demuth & Hahn, 2009; Ohno, 1970). Reductions in gene family size can also give rise to novel traits. For example, the colonization of highly specialized niches like oligotrophic caves (Gross et al., 2009; Protas et al., 2006; Yang et al., 2016) and toxic host plants (Good et al., 2014; Matsuo et al., 2007; McBride, 2007) is linked to rampant pseudogenization. Together, these observations suggest that some gene families evolve predictably in response to specific selection pressures. Yet compared to the rich and growing literature on genetic convergence at individual loci (Martin & Orgogozo, 2013), the repeatability and predictability of gene family evolution remains understudied (but see Beavan et al., 2023; Thomas et al., 2020).

The evolution of many gene families, defined here as groups of genes that share sequence and functional similarity from common ancestry (Dayhoff, 1976; Demuth & Hahn, 2009), is consistent with a birth‐death model where genes arise via duplication (gene gain) and lost via deletion or pseudogenization (gene loss) (Hughes & Nei, 1992; Nei & Rooney, 2005). When duplications and deletions evolve primarily through genetic drift, over time gene family sizes contract and expand via a process dubbed genomic drift (Nei, 2007; Nozawa et al., 2007). Overall, the stochastic birth‐death process of genomic drift (which differs from Nei's conceptual birth‐death model of gene family evolution; Hahn et al., 2005) sufficiently explains most gene family size distributions within genomes (Karev et al., 2002) and between species (Hahn et al., 2007).

But during an ecological shift, natural selection can influence birth‐death dynamics by driving the expansion or contraction of specific gene families. Thus, taxa adapted to a novel niche may have genomic evidence of selective maintenance for gene duplications or deletions. For example, if selection favors the retention of additional gene copies, novel gene duplicates will tend to persist in the genome, increasing the total number of genes. If the mutational mechanism that generates new duplicate genes is unequal crossing over during meiosis, these recently diverged paralogs will be arranged in tandem arrays across the genome (Zhang, 2003). Moreover, if duplicates experience positive selection for novel functions, they can have elevated amino acid substitution rates. Conversely, some genetic functions may become obsolete or even deleterious in the novel habitat. In this case, relaxed purifying selection or positive selection will cause some gene families to accumulate loss‐of‐function mutations at an accelerated rate (Go et al., 2005). After an ecological shift, impacted gene families will eventually reach a new equilibrium state where gene number returns to evolving primarily through negative selection and genomic drift, and pseudogenes fade into the genomic background as they accumulate neutral substitutions (Petrov et al., 1996; Petrov & Hartl, 1997, 1998). Thus, some footprints of adaptive changes in gene family size are likely ephemeral and best detected in lineages that *recently* shifted to a novel niche.

Arguably, the gene families most likely to evolve in response to niche shifts are those that mediate organismal interactions with their biotic and abiotic environments. These “environmentally responsive genes” include those with chemosensory (e.g., olfactory and gustatory receptors), detoxifying (e.g., cytochrome P450), and immunity (e.g., immunoglobulin and MHC) functions. To cope with constantly changing pressures, environmentally responsive genes tend to be characterized by elevated sequence diversity, duplication rates, substitution rates, and genomic clustering, as well as tissue‐ or temporal‐specific expression (Berenbaum, 2002) and limited pleiotropy (Arguello et al., 2016). Importantly, causal links between changes in environmentally responsive genes and adaptation to novel niches have been established for multiple taxa (Armisén et al., 2018; Després et al., 2007; Dobler et al., 2012; Luo et al., 2020; Matsuo et al., 2007; Sezutsu et al., 2013; Zhen et al., 2012).

With exceptionally diverse ecologies and an ever‐increasing availability of annotated genomes (Hotaling et al., 2021; i5K Consortium, 2013; Poelchau et al., 2015), insects are a powerful system for investigating the extent to which environmentally responsive gene families evolve predictably in response to ecological challenges. To date, at least two ecological transitions are hypothesized to have a predictable impact on gene family evolution in insect lineages. In plant‐feeding insects, the evolution of increased dietary specialization (i.e., smaller diet breadth) is associated with reduced chemosensory and detoxifying gene family sizes and, for intact genes, elevated rates of nonsynonymous substitutions (Calla et al., 2017; Comeault et al., 2017; Goldman‐Huertas et al., 2015; Good et al., 2014; McBride, 2007; McBride & Arguello, 2007) but see (Gardiner et al., 2008). In hymenopteran insects, eusociality is associated with expansions of the olfactory receptor family and contractions of the gustatory receptor family (Brand & Ramírez, 2017; McKenzie et al., 2016; Robertson & Wanner, 2006; Zhou et al., 2015; but see Fischman et al., 2011; Johnson et al., 2018). However, biased sampling in which insect lineages (especially *Drosophila* and apocritan Hymenoptera) and gene families (especially the olfactory receptor and cytochrome P450 gene families) are studied makes it difficult to draw general conclusions about evolutionary patterns. A better understanding of ecology and gene family size relationships requires a sampling of evolutionarily independent ecological transitions and functionally diverse gene families. To these ends, we characterize multiple environmentally responsive gene families in the genome of the redheaded pine sawfly, *Neodiprion lecontei* (Order: Hymenoptera; Family: Diprionidae).

*Neodiprion* is a genus of conifer‐feeding sawflies (Order: Hymenoptera; Family: Diprionidae). All species (~50 described to date; Linnen & Smith, 2012; Wallace & Cunningham, 1995) are restricted to host plants in the family Pinaceae; most are found exclusively on plants in the genus *Pinus*. Because most members of the genus are economically important pine tree pests (Arnett, 1993), the life histories of many *Neodiprion* species have been studied in great detail, providing extensive information on host use, behavior, morphology, and development (Atwood & Peck, 1943; Coppel & Benjamin, 1965; Knerer & Atwood, 1973). In addition to being well studied, *Neodiprion* are abundant in nature, can be reared and crossed under laboratory conditions (Knerer, 1984; Kraemer & Coppel, 1983; Linnen et al., 2018), and vary in many ecologically important traits (e.g., host range, larval color, grouping behavior, overwintering mode) (Knerer, 1993; Knerer & Atwood, 1973). In terms of host use, for example, different *Neodiprion* species specialize on different subsets of pine species with some species gaining the use of certain pine hosts and others losing the ability to use those same hosts (Linnen & Farrell, 2010). Together, these features make *Neodiprion* an excellent system for uncovering the molecular mechanisms and evolutionary processes that generate phenotypic variation. However, to realize the full potential of this promising model system, genomic resources such as annotated reference genomes are needed.

**FIGURE 1:**
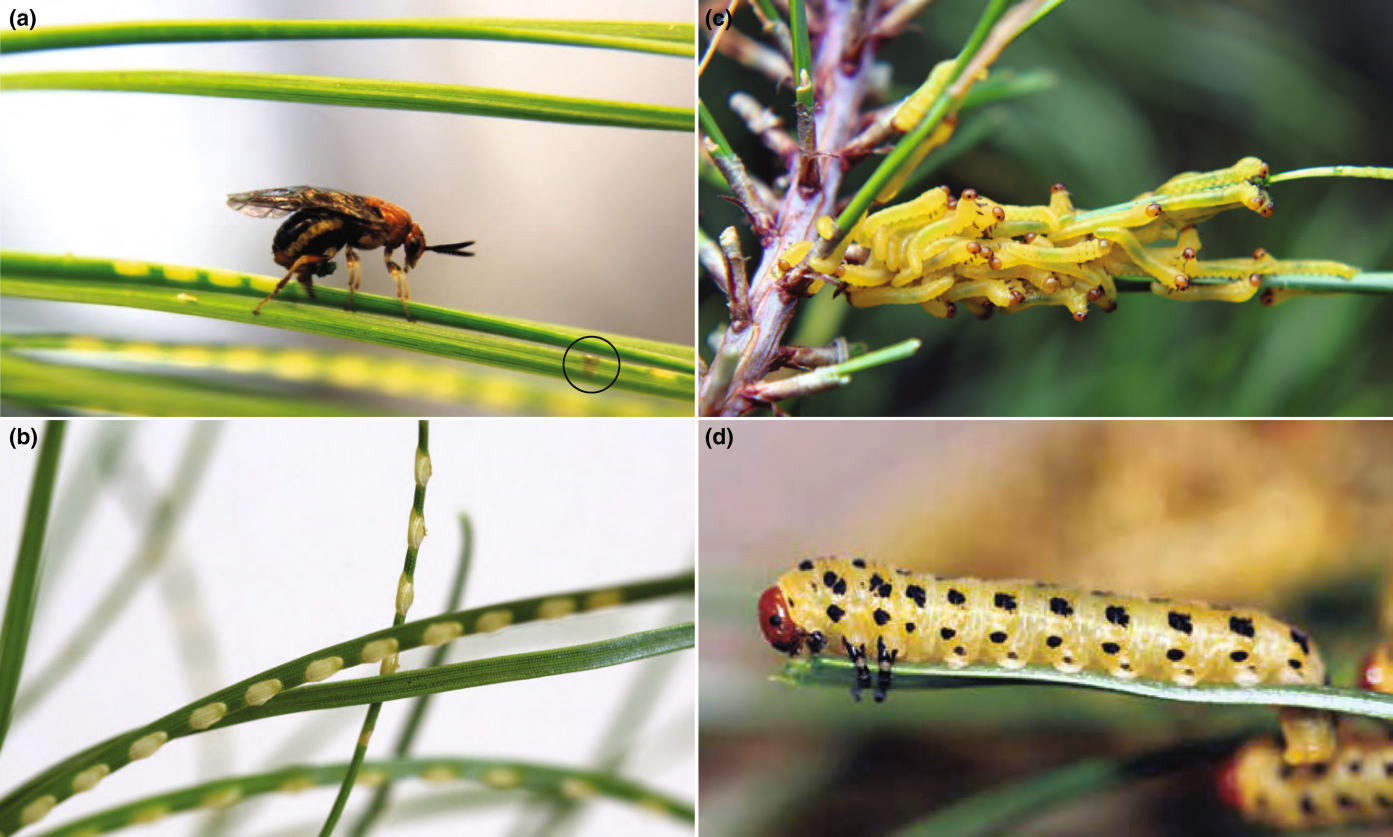
Diprionids transitioned from angiosperms to coniferous host plants and *N. lecontei* has multiple morphological and behavioral adaptations to *Pinus* foliage. (a) An egg‐laying *N. lecontei* female demonstrating several adaptations for ovipositing in thick, resinous pine needles, including: a robust saw‐like ovipositor (visible within the needle), a tendency to lay many closely spaced eggs per needle, and a tendency to cut resin‐draining slits on egg‐bearing needles (circled). (b) Throughout development, embryos are in close contact with living host tissue. Prior to hatching, *N. lecontei* eggs absorb water from the host, causing the eggs to swell and their pockets to open. (c) Early‐instar larvae have skeletonizing feeding behavior in which only the outer needle tissue is consumed, leaving the resinous interior intact. This strategy prevents small larvae from being overwhelmed by sticky resin. (d) Mid‐ and late‐instar larvae consume the entire pine needle. Larvae sequester pine resin in specialized pouches for use in self‐defense (All photos by R.K. Bagley).

Beyond the development of a novel model system, a draft genome for *N. lecontei* contributes a useful data point for comparative genomic analyses in two ways. First, although many assembled and annotated hymenopteran genomes are currently available, almost all are apocritan (ants, bees, and wasps, but see Falk et al., 2022; Michell et al., 2021; Oeyen et al., 2020; Robertson et al., 2018) for some recent exceptions). As the first draft genome from the symphytan family Diprioinidae, *N. lecontei* increases the ecological, behavioral, and taxonomic diversity of hymenopteran genomes for evaluating ecological correlates of gene family size and other aspects of genome evolution. Second, *N. lecontei* is an exemplar of an herbivorous insect lineage that underwent a drastic host shift. Sometime within the last 60 million years, the ancestor to extant diprionids transitioned from angiosperms to coniferous host plants in the family Pinaceae (Boevé et al., 2013; Peters et al., 2017). To defend against herbivores and pathogens, Pinaceae produce viscous oleoresin secretions with unique antimicrobial properties (Gershenzon & Dudareva, 2007; Trapp & Croteau, 2001). To manage these toxic and extraordinarily sticky resins, *N. lecontei* and related diprionids evolved specialized feeding and egg‐laying traits (Figure 1). Thus, beyond these traits, we hypothesize that pine specialization likely resulted in strong selection on multiple gene families, especially those involved in chemosensation, detoxification, and immune function.

Here, we describe the *N. lecontei* draft genome and compare it to other available hymenopteran and pine‐specialist genomes. To investigate gene families that may have contributed to pine adaptation, we manually annotated genes from five environmentally responsive gene families: olfactory receptor (OR), gustatory receptor (GR), odorant‐binding protein (OBP), cytochrome P450 (CYP), and antimicrobial peptide (AMP). For each gene family, we characterized (1) the number of genes, (2) the proportion of pseudogenized genes, (3) the extent of genomic clustering, (4) evolutionary relationships with hymenopteran orthologs, and (5) patterns of molecular evolution among recent paralogs. Based on these patterns, we identify candidate gene families that may have facilitated a shift from angiosperm feeding to pine‐feeding and evaluate how gene family size in Diprionidae compares to other manually annotated hymenopteran genomes.

## 2 RESULTS AND DISCUSSION

### 2.1 Genome assembly and annotation

#### 2.1.1 Sequencing and assembly

We sequenced one mate‐pair and two small‐insert Illumina libraries made from haploid male siblings (see Section 4). After read processing, we retained 268 billion PE100 reads with a combined read depth of 112× (Table A1). ALLPATHS‐LG (v47417) (Gnerre et al., 2011) produced a 239‐Mbp assembly consisting of 4523 scaffolds, with a scaffold N50 of 243 kbp (Table A2). Prior studies identified seven chromosomes in *N. lecontei* (Linnen et al., 2018; Maxwell, 1958; Smith, 1941; Sohi & Ennis, 1981), and flow cytometry estimated a genome size of 331 ± 9.6 Mbp (Harper et al., 2016); assuming that estimate of genome size is accurate, our assembly captured 72% of the genome. Overall, these metrics are comparable to other hymenopteran assemblies (Table A2).

To measure assembly completeness and artificial sequence duplication, we used CEGMA (Parra et al., 2007) and BUSCO (Simão et al., 2015). Both search the assembly for a set of single‐copy, conserved genes; however, the CEGMA software has been deprecated (http://korflab.ucdavis.edu/Datasets/cegma). Of the 248 CEGMA core eukaryotic genes, 90% aligned as complete, single copies and 8% aligned complete but duplicated. For BUSCO, we used the OrthoDB arthropod dataset, and out of 2675 groups 77% were complete, single copies and 3% were complete but duplicated. These metrics indicate the presence of artificial duplicate sequences, but otherwise the assembly was reasonably complete and suitable for annotation.

About 16% of the assembly consisted of repetitive elements, including 122 unknown transposable elements mostly unique to *N. lecontei*, and 212 other families of transposable elements and simple repeats (Table A3). This 16% corresponds to 12% of the actual 331‐Mb genome, of which we predict 28% is repetitive, suggesting that ~16% of the missing ~28% of the genome is repetitive content (Table A3). Overall, the repetitive element content is consistent with other sawfly genomes assembled from Illumina short reads (*Cephus cinctus*; Robertson et al., 2018), *Orussus abietinus* (Oeyen et al., 2020), but see *Athalia rosae* (Oeyen et al., 2020).

For automated gene prediction, we included the *N. lecontei* transcriptome and protein‐coding genes from *Atta cephalotes* (OGSv1.2), *Acromyrmex echinatior* (OGSv3.8), *Apis mellifera* (OGSv3.2), *Athalia rosae* (OGSv1.0), and *Nasonia vitripennis* (OGSv1.0) to guide annotation. The official gene set (OGSv1) had 12,980 gene models while the transcriptome had an average of 26,000 transcripts per tissue (Table A4). The number of *N. lecontei* gene annotations is on the lower end for Hymenoptera, where gene number ranges from about 11,000 to 24,000 (Table A2).

### 2.2 Size, genomic arrangement, and evolutionary history of environmentally responsive gene families

#### 2.2.1 Olfactory receptors

**FIGURE 2:**
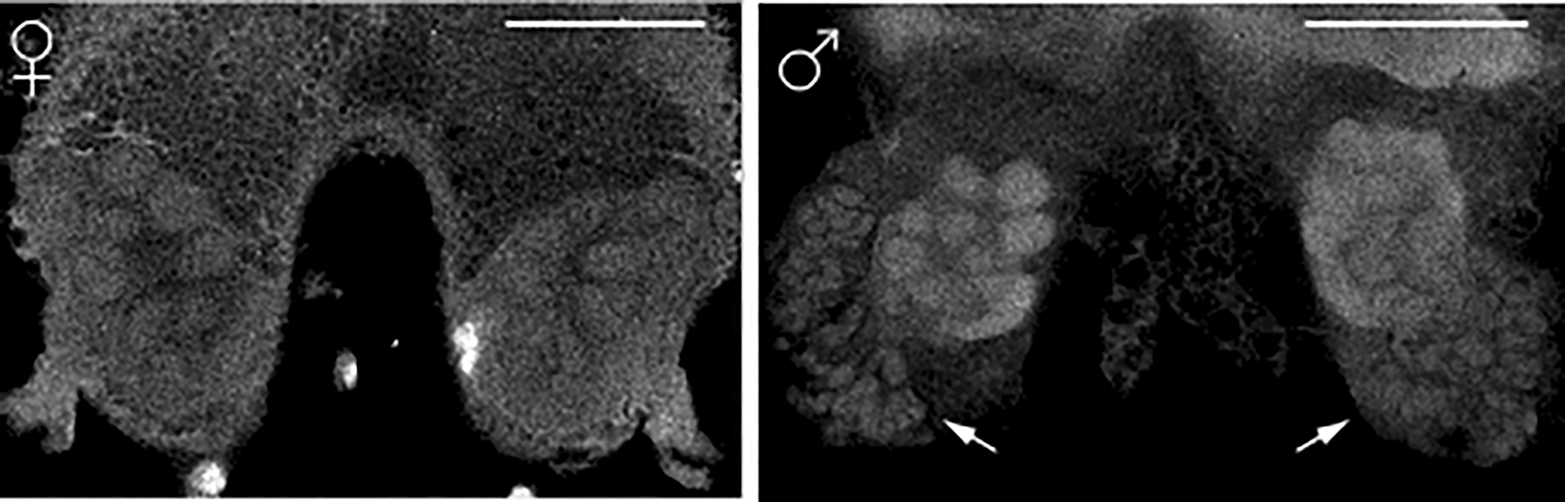
Optical sections through the antennal lobes of adult female (left) and male (right) *N. lecontei*. White arrows indicate regions of male‐specific synaptic clusters. Scale bars = 500 μm.

**FIGURE 3:**
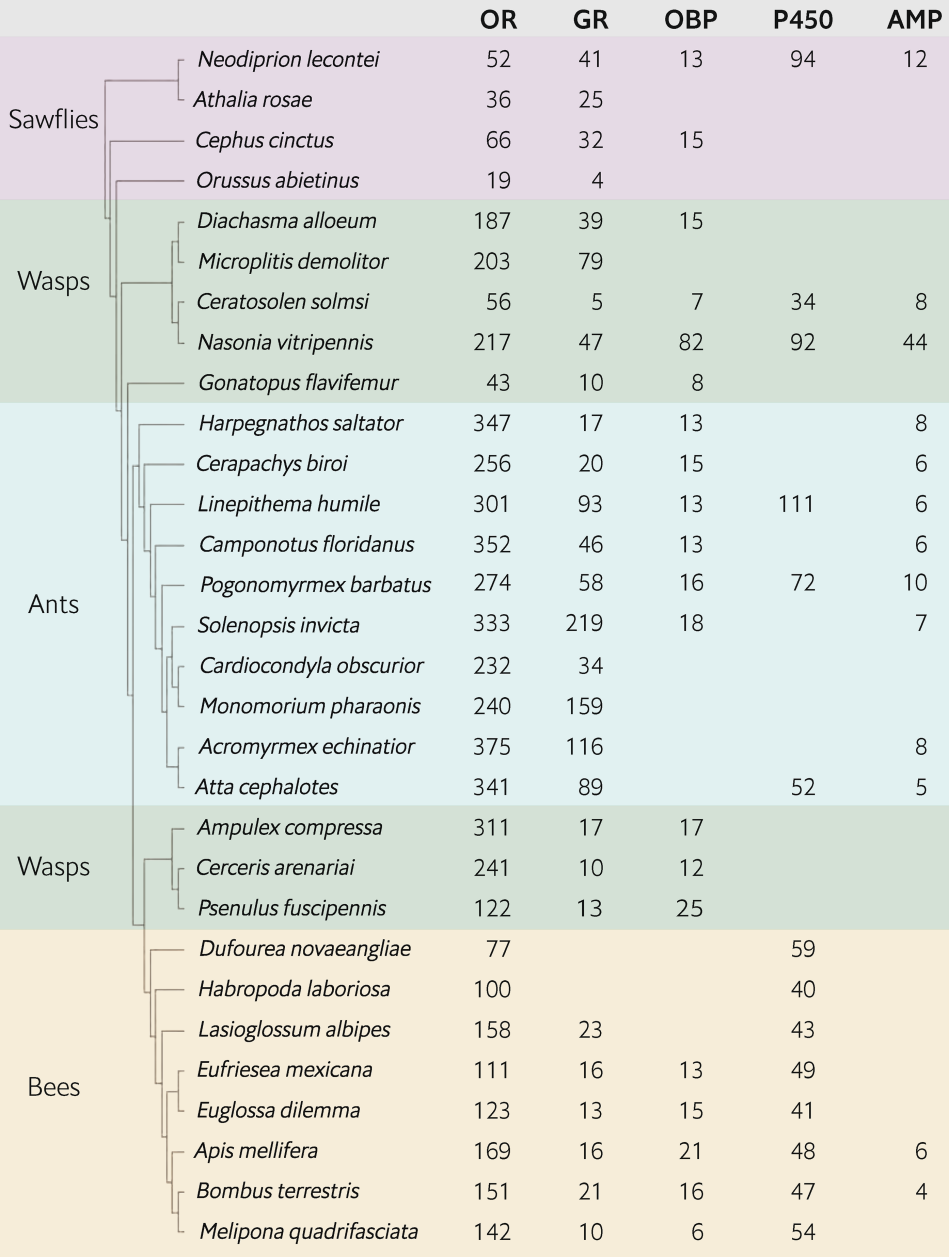
Number of intact genes in hymenopteran genomes for each of five environmentally responsive gene families. Phylogenetic relationships are as in Moreau et al. (2006), Hedtke et al. (2013), Roux et al. (2014), Brand et al. (2017), Branstetter et al. (2018), Peters et al. (2017). Branch lengths are arbitrary (i.e., do not reflect substitution rates or time). Gene family abbreviations: AMP, antimicrobial protein; CYP, cytochrome P450; GR, gustatory receptor; OBP, odorant‐binding protein; OR, olfactory receptor.

The OR gene family had 56 genes total, including the co‐receptor *Orco*; one gene contained stop codons and three were partial annotations, leaving 52 genes intact (Table 1). In *D. melanogaster*, most olfactory sensory neurons (OSNs) express a single OR (along with *Orco*). Furthermore, OSNs that express a particular OR all converge on the same glomerulus in the antennal lobe (Couto et al., 2005; Gao et al., 2000; Vosshall et al., 2000; but see Fishilevich & Vosshall, 2005), resulting in a general one‐to‐one anatomy between the number of ORs and the number of glomeruli, correspondence also observed in the hymenopteran European honeybee (*Apis mellifera*; Robertson & Wanner, 2006). Based on these studies and examination of adult male and adult female *N. lecontei* antennal lobes, we expected to find a minimum of 49 functional ORs (Figure 2, Table A5). The observed size of the *Neodiprion lecontei* OR gene family exceeds this minimum, is comparable to other herbivorous sawflies, and is much smaller than that of ants, bees, and many wasps (Figure 3, Table A8). Thus, our data are consistent with the hypothesis that eusocial hymenopterans have unusually large OR families, possibly due to selection stemming from complex chemical communication (LeBoeuf et al., 2013; Robertson & Wanner, 2006; Zhou et al., 2015; but see Brand & Ramírez, 2017; Roux et al., 2014).

**TABLE 1:** Summary of gene family size, genomic clustering, and patterns of molecular evolution for five environmentally responsive gene families.

Gene family	Gene family size					Genomic clustering		Molecular evolution		
	Intact genes	Partial	Pseudo	Total genes	Prop. Pseudo	Prop. in clusters^a^	Largest cluster	*Neodiprion*‐specific clades^b^	Significant branch tests^c^	Significant site tests^d^
OR	52	3	1	56	0.02	0.59	8	3	2	1
GR	41	2	2	44^e^	0.05	0.76	10	3	2	1
OBP	13	0	0	13	0	0.38	3	0	n/a	n/a
CYP (all)	93	2	12	107	0.11	0.66	16	5	2	0
CYP2 clan	9	0	0	9	0	0.33	2	0	0	0
CYP3 clan	47	0	8	55	0.15	0.81	16	4	2	0
CYP4 clan	27	2	4	33	0.12	0.55	3	1	0	0
mito CYP clan	10	0	0	10	0	0.50	3	0	0	0
AMP	21	0	0	21	0	0.95	15	?^f^	0	0

Abbreviations: AMP, antimicrobial peptide genes; CYP, cytochrome P450 genes (“clans” refer to four major clades of CYPs present in insects); GR, gustatory receptor genes; OBP, odorant‐binding protein genes; OR, olfactory receptor genes.

^a^ Calculated as: (number of genes in clusters of 2 or more)/(genes for which clustering could be evaluated).

^b^ Defined as monophyletic clusters of 4 or more *Neodiprion* paralogs in an amino acid phylogeny constructed with gene annotations from *Neodiprion*, select Hymenoptera (including additional sawflies), and *Drosophila melanogaster*.

^c^ To be counted, clades had to reject both 1‐ratio and fixed‐ratio models in dN/dS branch tests (see Table 2).

^d^ To be counted, clades had to reject both M7 and M8a models in dN/dS site tests (see Table 2).

^e^ One gene was both a partial annotation and a pseudogene.

^f^ Low bootstrap support precluded the identification of *Neodiprion*‐specific clades.

**FIGURE 4:**
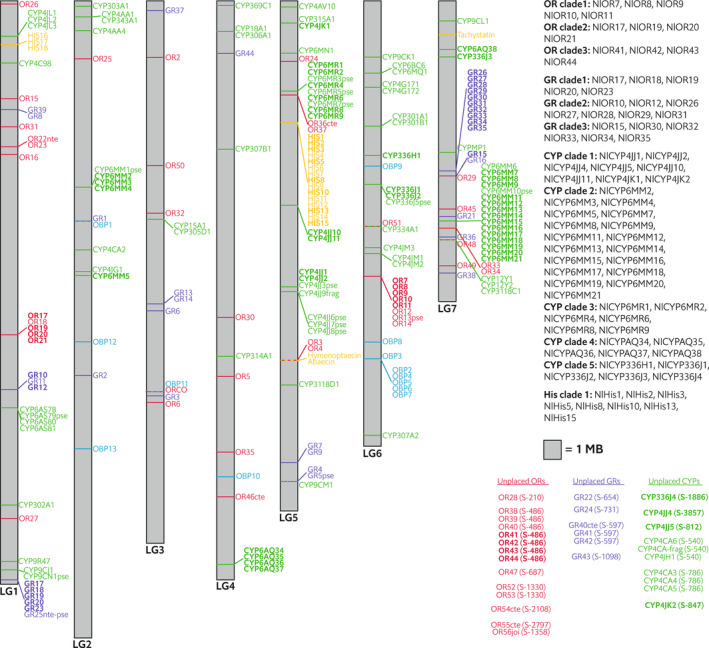
Position of genes belonging to five environmentally responsive gene families along seven *N. lecontei* linkage groups. Linkage groups (LG) are drawn to scale and ordered as in the linkage‐group anchored assembly described in Linnen et al. (2018) (GenBank accession numbers are as follows: LG1 = CM009916.1; LG2 = CM009917.1; LG3 = CM009918.1; LG4 = CM009919.1; LG5 = CM009920.1; LG6 = CM009921.1; LG7 = CM009922.1). Gene family abbreviations are as in Figure 3. Each gene family is represented by a different color. Horizontal lines indicate the approximate locations of genes within LG; diagonal lines that connect to horizontal lines are used to highlight groups of genes that met our clustering criteria. Genes that were found on scaffolds that have not been placed on linkage groups are indicated on the bottom left, with abbreviated scaffold names given in parentheses (e.g., S‐210 = scaffold_210 = LGIB01000210.1 in the assemblies available on NCBI).

**FIGURE A1:**
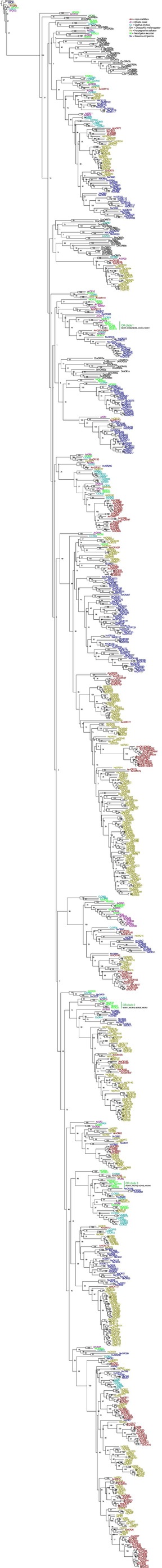
Select Hymenoptera olfactory receptor gene family tree (amino acid). Included genes were manually annotated, intact (not partial or pseudogene), with a minimum length of 350 AA.

**FIGURE A2:**
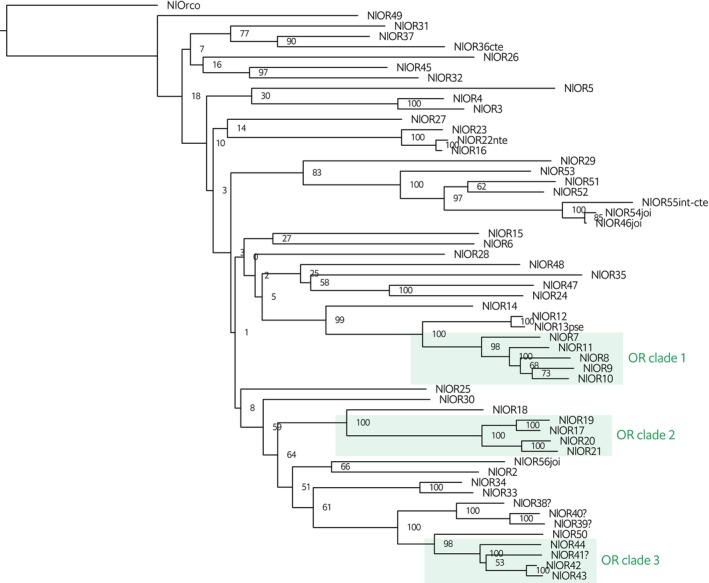
*Neodiprion lecontei* olfactory receptor gene family tree (cDNA). Genes were manually annotated and all annotations (intact, pseudogene, and partial) were included.

59% of ORs were in genomic clusters of two or more genes (Figure 4), a low proportion compared to many other hymenopteran OR families (Brand & Ramírez, 2017; McKenzie et al., 2016; Robertson & Wanner, 2006; Zhou et al., 2015). A phylogenetic analysis of OR protein sequences from *Neodiprion lecontei*, two other sawfly species, three apocritan Hymenoptera, and *D. melanogaster* identified three *Neodiprion*‐specific clades of at least four genes (Table 2, Figure A1); these were also recovered in a phylogenetic analysis of *Neodiprion* OR cDNA sequences (Figure A2). All these clades were found in genomic clusters mixed with other OR genes (Figure 4). For the *Neodiprion*‐specific OR clades (and other *Neodiprion*‐specific clades, see below), we used the *Neodiprion* gene family cDNA tree, the codeml program in the PAML package (Yang, 2007), and likelihood‐ratio tests to ask: (1) for the focal OR clade if the ratio of nonsynonymous to synonymous substitution rates (dN/dS or *ω*) differed from the rest of the *Neodiprion* OR gene family and if so, whether the clade exhibited evidence of non‐neutral evolution (*ω* ≠ 1) (branch tests) and (2) within *Neodiprion*‐specific clades whether ω differed among amino acids across sites and if so, which sites exhibited evidence of positive selection (site tests). Of the *Neodiprion*‐specific OR clades, clade 2 had significant branch tests and clade 3 had significant branch but ambiguous site tests (Table 2). Significant branch tests without significant site tests can occur when the clade's branch had an elevated ω value but site‐specific selection (if present) acted on sites that varied across paralogs or if insufficient sequence changes have occurred for detectability.

**TABLE 2:** Likelihood‐ratio tests (LRTs) of positive selection on *Neodiprion*‐specific clades (branch models) and on amino acid sites within these clades (site models).

Clade names^a^	*n* ^b^	Model comparison^c^	LRT statistic^d^	df	*p*‐Value^e^	*ω* (dN/dS)^f^
Olfactory receptor						
OR clade 1	5	M8 vs. M7	1.80	2	.41	
		M8 vs. M8a	1.95	1	.16	
		2‐ratio vs. 1‐ratio	0.17	1	.68	
		2‐ratio vs. 2‐ratio neutral	1.70	1	.38	
OR clade 2	4	M8 vs. M7	0.30	2	.86	
		M8 vs. M8a	0.53	1	.47	
		2‐ratio vs. 1‐ratio	6.92	1	**8.53 e‐03**	(0.26, 0.01)
		2‐ratio vs. 2‐ratio neutral	16.55	1	**9.50 e‐05**	
OR clade 3	4	M8 vs. M7	5.69	2	.06	
		M8 vs. M8a	4.42	1	**.04**	
		2‐ratio vs. 1‐ratio	7.93	1	**4.85 e‐03**	(0.26, 0.07)
		2‐ratio vs. 2‐ratio neutral	21.01	1	**9.14 e‐06**	
Gustatory receptor						
GR clade 1	5	M8 vs. M7	39.14	2	**3.16 e‐09**	84(E), 86(S), 154(N), 288(S), 313(S)^g^
		M8 vs. M8a	34.96	1	**3.37 e‐09**	
		2‐ratio vs. 1‐ratio	14.79	1	**1.20 e‐04**	(0.39, 0.01)
		2‐ratio vs. 2‐ratio neutral	27.81	1	**2.68 e‐07**	
GR clade 2	7	M8 vs. M7	2.21	2	.33	
		M8 vs. M8a	1.16	1	.28	
		2‐ratio vs. 1‐ratio	0.00	1	.95	
		2‐ratio vs. 2‐ratio neutral	0.65	1	.42	
GR clade 3^h^	6	M8 vs. M7	6.92	2	**.03**	
		M8 vs. M8a	1.78	1	.18	
		2‐ratio vs. 1‐ratio	3.56	1	.06	(0.39, 0.11)
		2‐ratio vs. 2‐ratio neutral	7.21	1	**.01**	
Cytochrome P450						
Clade 1 (CYP4 clan)	8	M8 vs. M7	0.62	2	.74	
		M8 vs. M8a	0.87	1	.35	
		2‐ratio vs. 1‐ratio	0.66	1	.42	
		2‐ratio vs. 2‐ratio neutral	0.09	1	1.00	
Clade 2 (CYP3 clan)	18	M8 vs. M7	0.00	2	1.00	
		M8 vs. M8a	0.00	1	1.00	
		2‐ratio vs. 1‐ratio	2.08	1	.15	
		2‐ratio vs. 2‐ratio neutral	0.08	1	1.00	
Clade 3 (CYP3 clan)	6	M8 vs. M7	7.15	2	**.03**	
		M8 vs. M8a	0.65	1	.42	
		2‐ratio vs. 1‐ratio	6.33	1	**.01**	(0.16, 0.01)
		2‐ratio vs. 2‐ratio neutral	14.26	1	**3.18 e‐04**	
Clade 4 (CYP3 clan)	5	M8 vs. M7	0.00	2	1.00	
		M8 vs. M8a	0.15	1	.70	
		2‐ratio vs. 1‐ratio	0	1	.96	
		2‐ratio vs. 2‐ratio neutral	0.94	1	.33	
Clade 5 (CYP3 clan)	5	M8 vs. M7	0.00	2	1.00	
		M8 vs. M8a	0.00	1	1.00	
		2‐ratio vs. 1‐ratio	5.33	1	**.02**	(0.16, 0.01)
		2‐ratio vs. 2‐ratio neutral	12.29	1	**9.12 e‐04**	
Hisnavicin (Antimicrobial peptide)						
Clade 1	15	M8 vs. M7	2.39	2	.67	
		M8 vs. M8a	0.00	1	1.00	
		2‐ratio vs. 1‐ratio	7.91	1	**4.92 e‐03**	(0.08 0.47)
		2‐ratio vs. 2‐ratio neutral	1.00	1	.64	

^a^ Clade names are as in Figures A1 through https://doi.org/10.5061/dryad.n8pk0p320.

^b^ Putatively functional genes. Pseudogenes and partial annotations were excluded from analysis.

^c^ Site models unshaded; M7 and M8a do not allow for positive selection. Branch models shaded; 1‐ratio estimates a single *ω* value for all branches, 2‐ratio estimates a separate *ω* value for the foreground branch, 2‐ratio neutral fixes *ω* = 1 for all branches.

^d^ Likelihood‐ratio test statistic, calculated as twice the difference in model log likelihoods (2*(∆LRT)).

^e^ Bolded values are significant at critical value 0.05.

^f^ 2‐ratio model values (foreground branch, rest of tree).

^g^ Amino acid sites under positive selection (from M8 Bayes Empirical Bayes analysis *p* > 95%).

^h^ Reported values are from analyses without NlGR16.

#### 2.2.2 Gustatory receptors

The GR gene family had 44 genes total; two genes contained stop codons, two were partial annotations (one annotation was both partial and pseudogenized), and 41 were intact (Table 1). In contrast to the OR gene family, the *N. lecontei* GR family size was larger than other sawflies, and considerably larger than most bees (Figure 3, Table A8); still, the largest hymenopteran GR families are among several ant species. Overall, the patterns of GR family size variation in Hymenoptera do not appear associated with eusociality, as suggested by (Robertson & Wanner, 2006). But given the pronounced variation among taxa, it is possible that other ecological transitions—like changes in diet breadth or specialization on specific niches—have favored GR family expansions or contractions in certain hymenopteran lineages. Additional data (i.e., ecological character states and GR annotations from diverse lineages) are needed to test this hypothesis.

**FIGURE A3:**
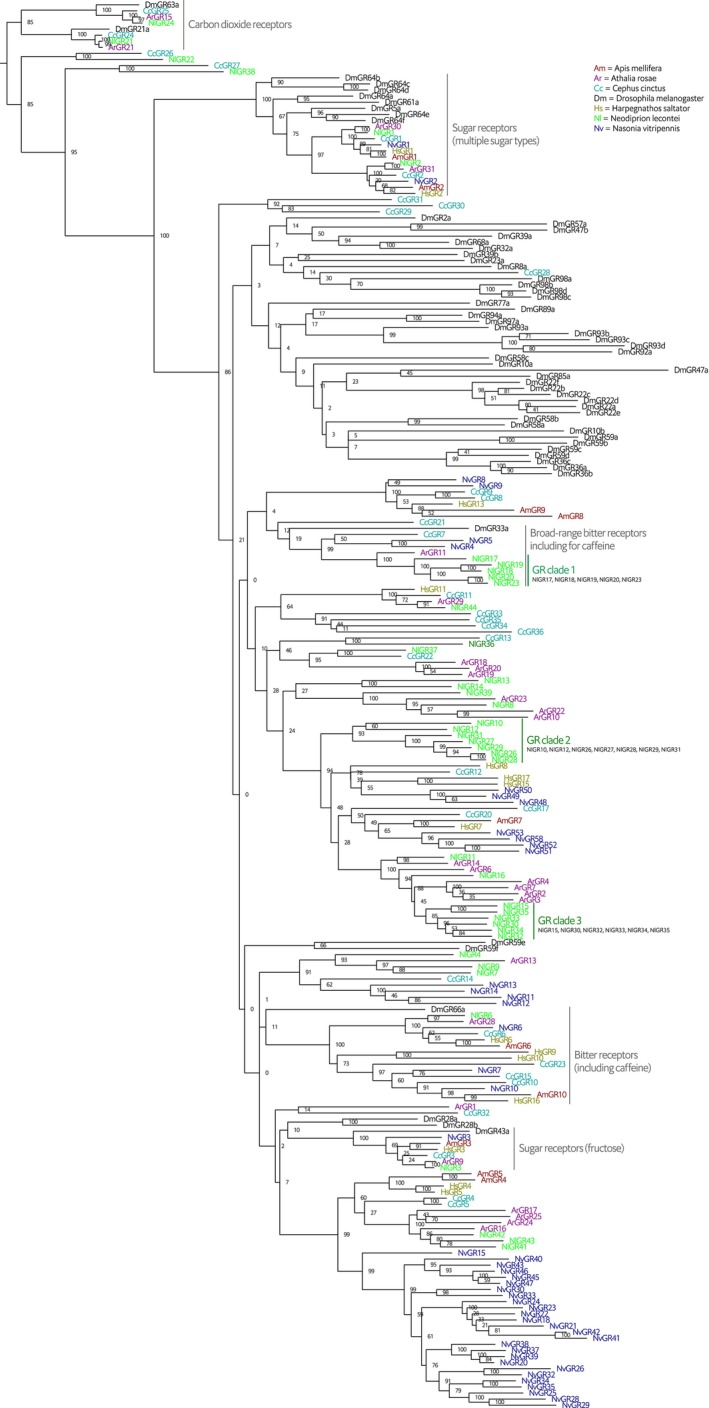
Select Hymenoptera gustatory receptor gene family tree (amino acid). Included genes were manually annotated, intact (not partial or pseudogene), with a minimum length of 350 AA.

**FIGURE A4:**
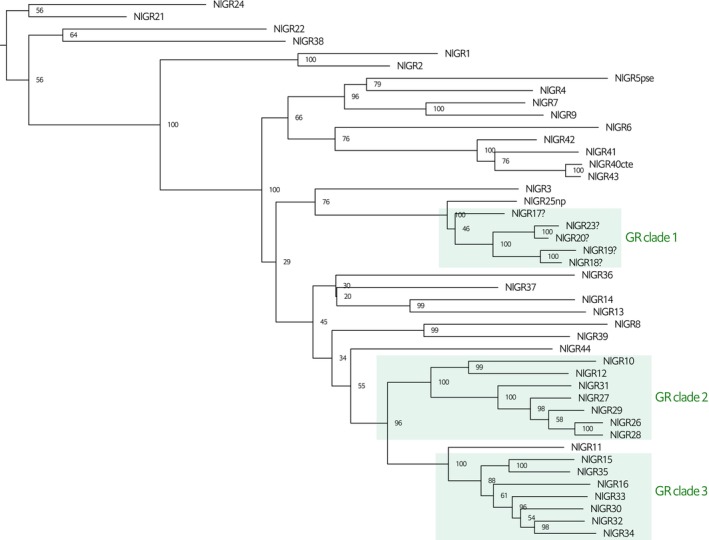
*Neodiprion lecontei* gustatory receptor gene family tree (cDNA). Genes were manually annotated and all annotations (intact, pseudogene, and partial) were included.

76% of the GRs that could be placed on chromosomes were in genomic clusters (Figure 4) including the three *Neodiprion*‐specific GR clades (Table 2, Figures A3 and A4). GR clade 3 had a discrepancy between the hymenopteran GR protein tree and *Neodiprion* cDNA tree where NlGR16 was part of the clade in the *N. lecontei* cDNA tree but not in the hymenopteran amino acid tree; analyses with and without GR16 had similar results. Genomic clustering was evident in all three *Neodiprion*‐specific GR clades; however, some of the genes in clades 2 and 3 were clustered together, suggesting shared ancestry from an older duplication event (also see Figure A3). Clade 1 had significant values for all four tests of selection (branch and site), clade 2 lacked evidence for positive selection, and clade 3 had evidence of branch and possibly site positive selection (Table 2). Notably, GR Clade 1 (which had strong evidence for branch and site positive selection) is an expansion of five paralogs orthologous to *DmGR33a*, a co‐receptor required for caffeine detection. In *Drosophila*, three GRs are known to be required for detecting caffeine (a bitter‐tasting deterrent compound): *DmGR93a*, *DmGR66a*, *DmGR33a* (Lee et al., 2009; Moon et al., 2006, 2009). *DmGR93a* is a fine‐tuned receptor more specific for caffeine while *DmGR66a* and *DmGR33a* are broad tuned for a wide range of avoidance compounds (Shim et al., 2015). Because *N. lecontei* has a paralogous expansion of only the broadly tuned *DmGR33a* ortholog and not the two other caffeine co‐receptors, it may be the case that *N. lecontei* does not have an increased sensitivity to caffeine but rather an increased sensitivity to a broader range of bitter compounds.

GR orthologs were also found for sugar receptors *DmGR5a* (trehalose) (Dahanukar et al., 2001), *DmGR43a* (fructose) (Miyamoto et al., 2012), and *DmGR64a‐f* (multiple sugars) (Slone et al., 2007) plus carbon dioxide receptors *DmGR21a* and *DmGR63a* (Jones et al., 2007) (Figure A3). Orthologs to these carbon dioxide receptors have not been found in Apocrita (Robertson & Kent, 2009) or the parasitoid sawfly *Orussus abietinus* (Oeyen et al., 2020) but seem to be preserved in phytophagous Symphyta like *N. lecontei* and *Cephus cinctus* (Robertson et al., 2018).

#### 2.2.3 Odorant‐binding protein

**FIGURE A5:**
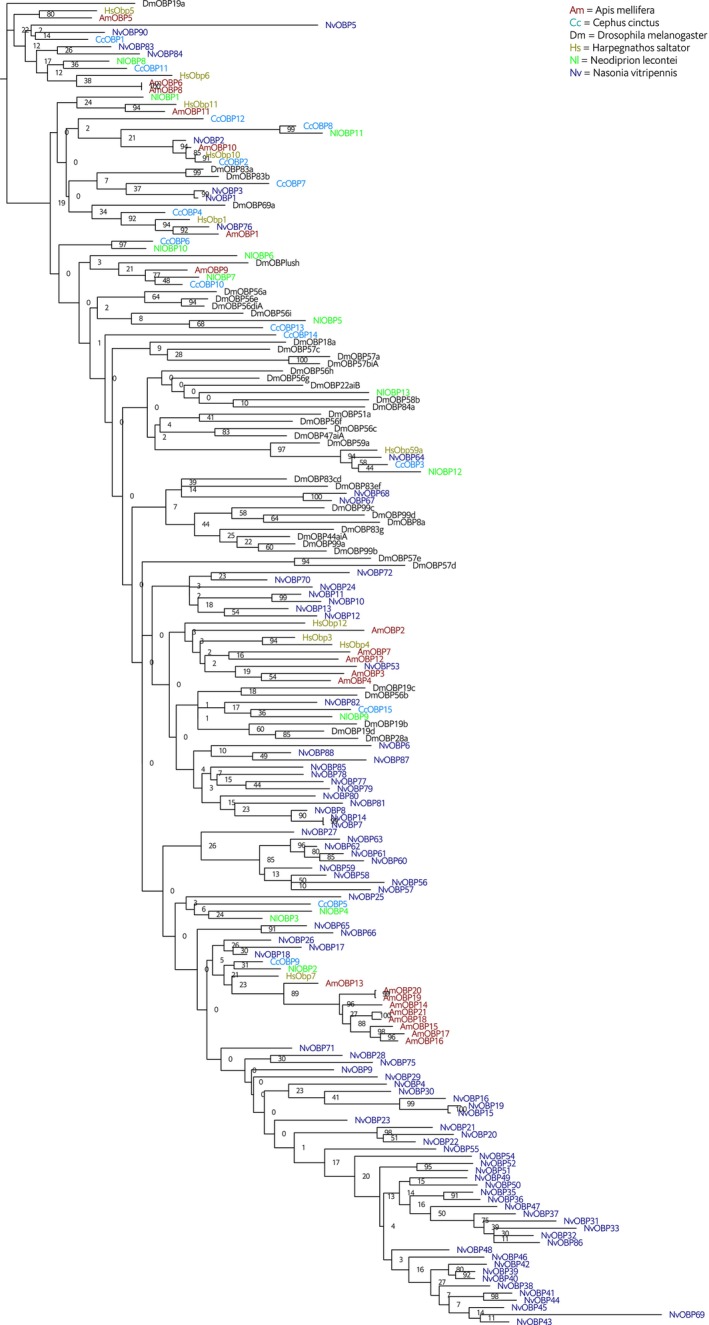
Select Hymenoptera odorant‐binding protein gene family tree (amino acid). Included genes were manually annotated, intact (not partial or pseudogene), with a minimum length of 100 AA.

**FIGURE A6:**
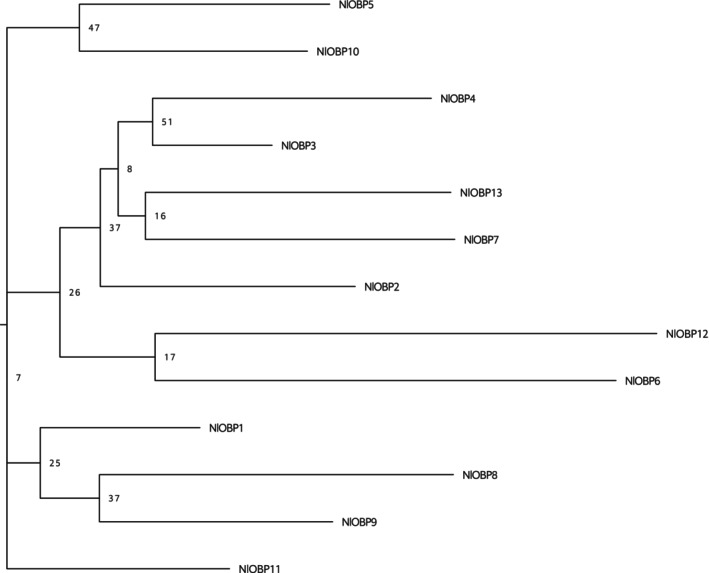
*Neodiprion lecontei* odorant‐binding protein gene family tree (cDNA). Genes were manually annotated and all annotations (intact, pseudogene, and partial) were included.

The OBP gene family had 13 intact genes total; pseudogenized or partial annotations were not identified (Table 1). This family size is on par with other hymenopterans, but it is important to note that insect OBP annotations are sparse compared to OR and GR annotations (Figure 3, Table A8) so interesting family size variation may be overlooked. It is apparent, however, that except for an unusually large family in *Nasonia vitripennis*, family size is much less variable across taxa, suggesting that OBP gene family evolution is more constrained (Vieira et al., 2007; Vieira & Rozas, 2011). Unlike the other chemosensory gene families in this study, phylogenetic grouping failed to identify any *Neodiprion*‐specific OBP clades (Figure A5) and only 38% of OBP genes were in genomic clusters, including a five gene cluster on chromosome 6 (Figure 4) that was not monophyletic (Figure A6). OBP lineage‐specific expansions and genomic clustering are common in insects including Hymenoptera (e.g., Forêt & Maleszka, 2006; Jiang et al., 2017; Vieira et al., 2012; Vogt et al., 2015). We note, however, that our OBP phylogenies had low bootstrap support (Figures A5 and A6), making it difficult to infer paralogous relationships.

To compare, the wheat‐stem sawfly *Cephus cinctus* also had a similar gene family size (15 genes) and lack of species‐specific clades (Figure A5), but 63% of genes were in genomic clusters (Robertson et al., 2018). Two nonmutually exclusive explanations for the lack of *OBP* clustering in the *N. lecontei* genome are: (1) existing genes are in genomic regions with low duplication rates (Langley et al., 2012) and/or (2) OBP gene duplications tend to be removed by purifying selection.

#### 2.2.4 Cytochrome P450

**FIGURE A7:**
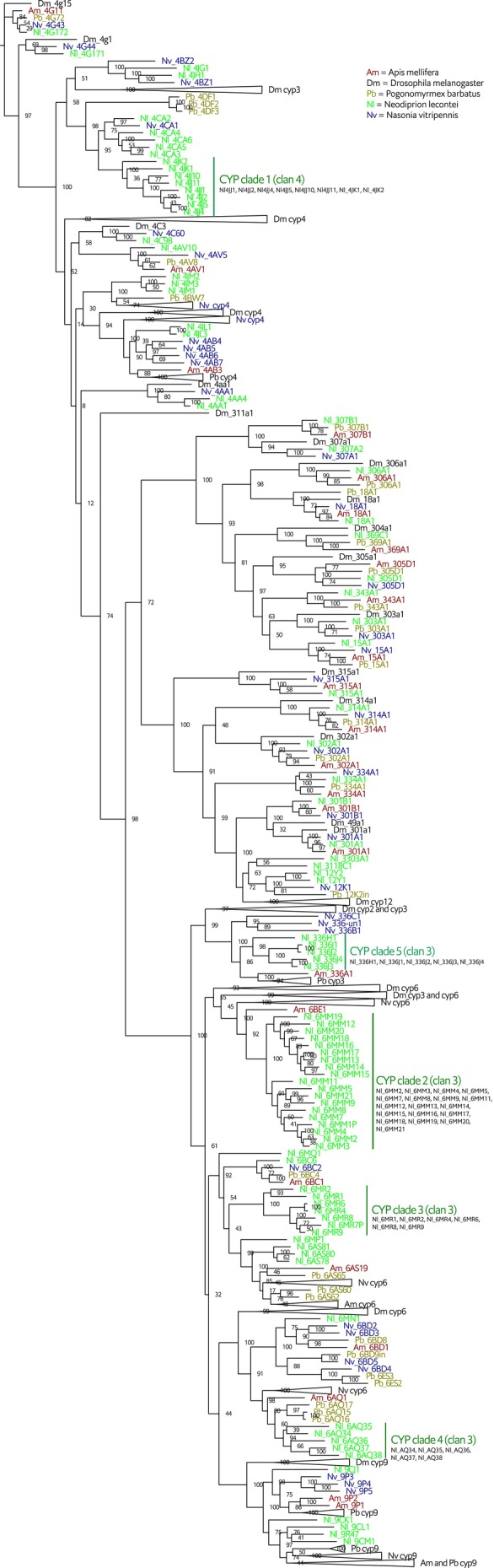
Select Hymenoptera cytochrome P450 gene family tree (amino acid). Included genes were manually annotated, intact (not partial or pseudogene), with a minimum length of 350 AA.

**FIGURE A8:**
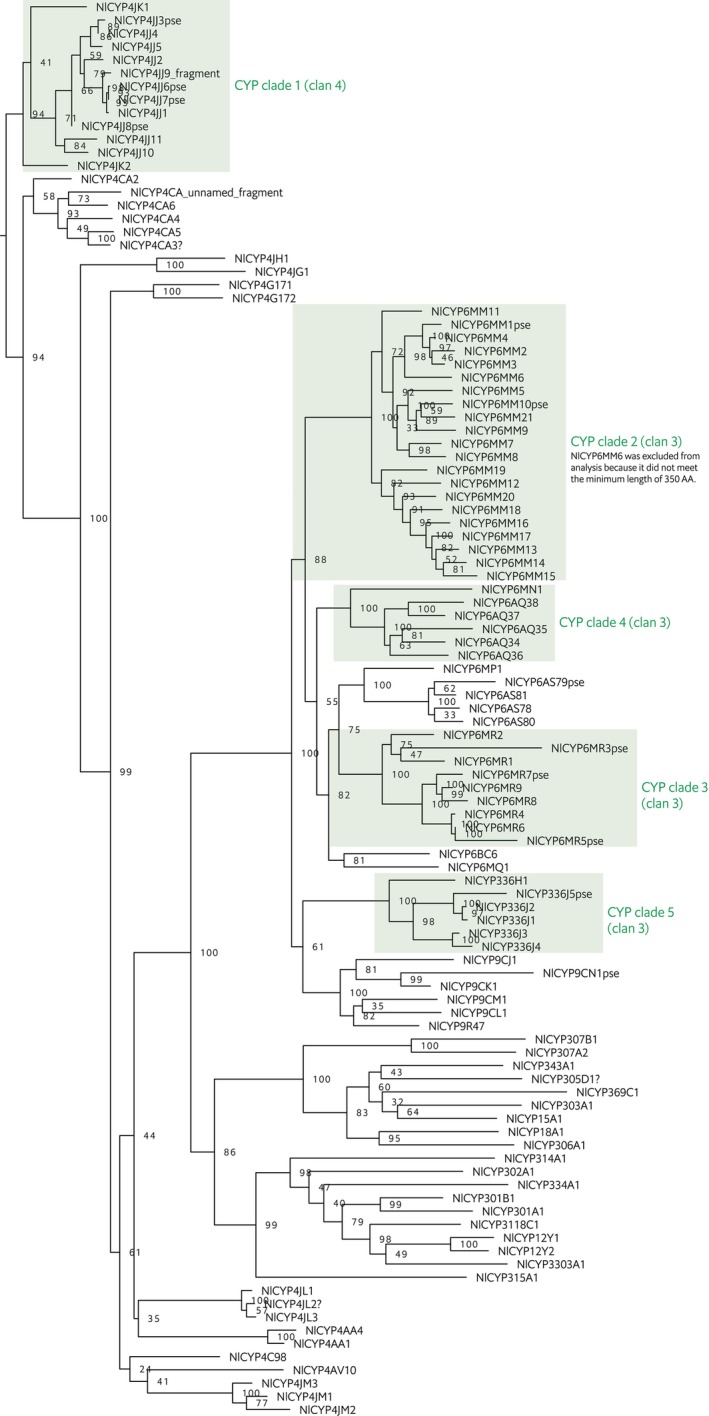
*Neodiprion lecontei* cytochrome P450 gene family tree (cDNA). Genes were manually.

The CYP gene family had 107 genes total; 12 genes contained stop codons, one was a partial annotation, and 94 were intact (Table 1). The number of intact CYP genes in *N. lecontei* is higher than many wasp and ant species and considerably higher compared to bee species (Figure 3, https://doi.org/10.5061/dryad.n8pk0p320). Thus, a reduced complement of CYPs may be unique to bees. But without CYP annotations from other sawflies, it is unclear if *N. lecontei* has an unusually large family size for a hymenopteran. In insects, CYPs belong to four major clades, referred to as clans (Feyereisen, 2012). When split by clan, the CYP2 clan had nine intact genes; the CYP3 clan had 47 intact genes and eight pseudogenes; the CYP4 clan had 28 intact genes, four pseudogenes, and one partial gene, and the mitochondrial CYP clan had 10 intact genes (Table 1, Figures A7 and A8). Across all CYPs, 66% were in genomic clusters (Figure 4). Looking at the four major clans separately, the percentage of clustered genes were: 33% for CYP2, 81% for CYP3, 55% for CYP4, and 50% for mitochondrial CYP (Figure 4).

The CYP gene family had five *Neodiprion*‐specific clades with at least four genes (Figures A7 and A8), four of which were in the CYP3 clan. Of these, CYP clades 3 (CYP6 subfamily) and 5 (CYP336) had evidence of branch‐specific, but not site‐specific, positive selection (Table 2). Several studies to date suggest that members of the CYP3 clan—and the CYP6 subfamily in particular—play an important role in detoxifying pesticides and host‐plant allelochemicals (Feyereisen, 2012).

In clan 2, *CYP303A1* is involved in mechano‐ and chemosensory bristle development (Willingham & Keil, 2004). It is found across winged insects and has a highly conserved length of 498 + 4 amino acids except in Hymenoptera, where orthologs have an insertion that increases the length to 562 amino acids in *A. mellifera* and 587 in *N. vitripennis* (Dermauw et al., 2020). The *N. lecontei* ortholog had 578 amino acids, suggesting that the *CYP303A1* insertion is ancestral in Hymenoptera.

Orthologs were found for all the Halloween genes (CYP2 and mitochondrial clans) of the 20‐hydroxy ecdysone biosynthesis pathway: *CYP302A1* (disembodied), *CYP306A1* (phantom), *CYP307A2* (spookier), *CYP307B1* (spookiest), *CYP314A1* (shade), *CYP315A1* (shadow), and *CYP18A1*, which turns over 20‐hydroxy ecdysone (Feyereisen, 2011; Guittard et al., 2011; Qu et al., 2015; Rewitz et al., 2007). The juvenile hormone biosynthesis gene *CYP15A1* was also present (Helvig et al., 2004). Finally, *N. lecontei* had orthologs for the two CYP4G enzymes that synthesize the cuticular hydrocarbons used as external waterproof coating (Qiu et al., 2012).

#### 2.2.5 Immunity

**FIGURE A9:**
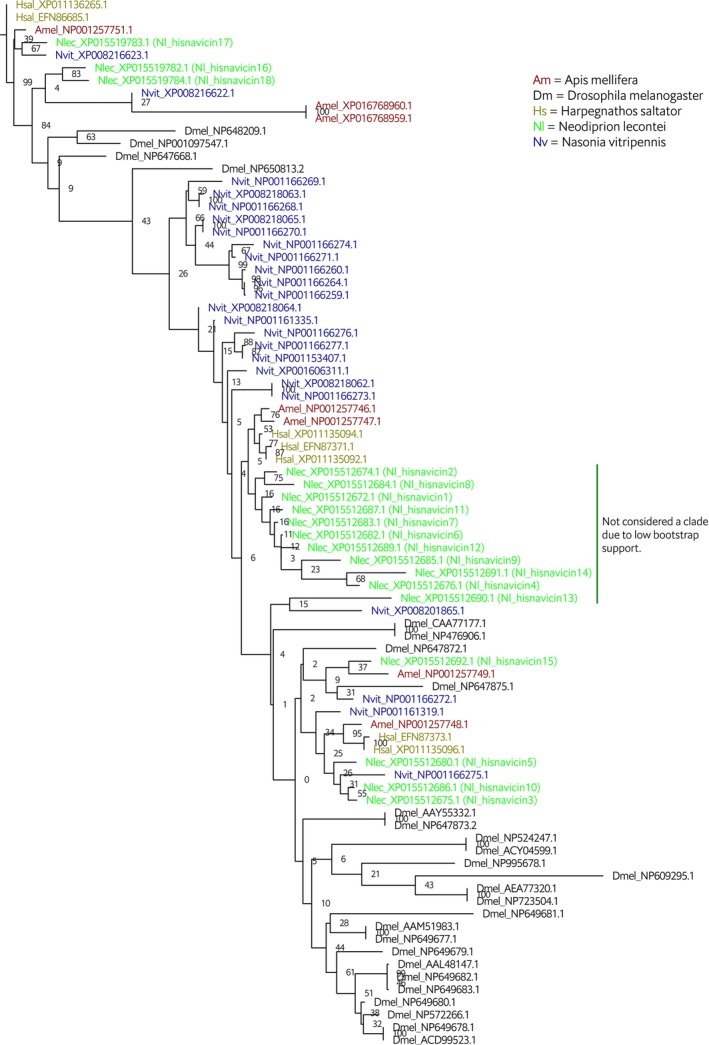
Select Hymenoptera hisnavicin gene family tree (amino acid). Included genes were intact (not partial or pseudogene).

**FIGURE A10:**
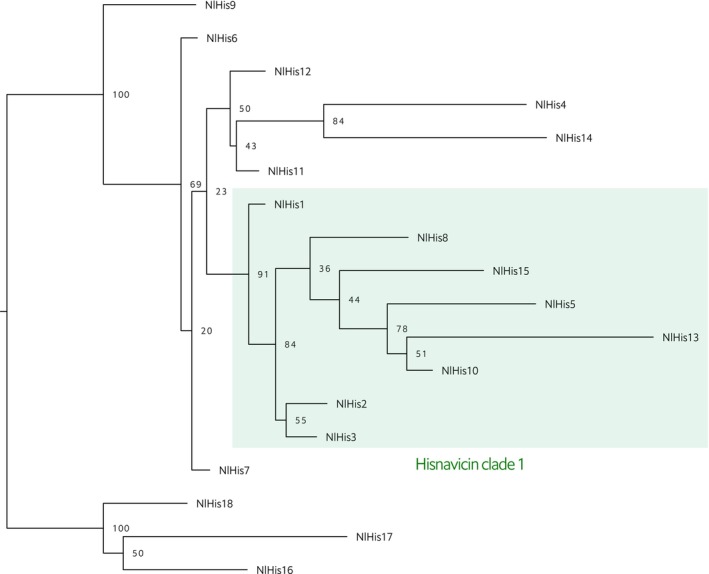
*Neodiprion lecontei* hisnavicin gene family tree (cDNA). Genes were manually annotated and all annotations (intact, pseudogene, and partial) were included.

**FIGURE A11:**
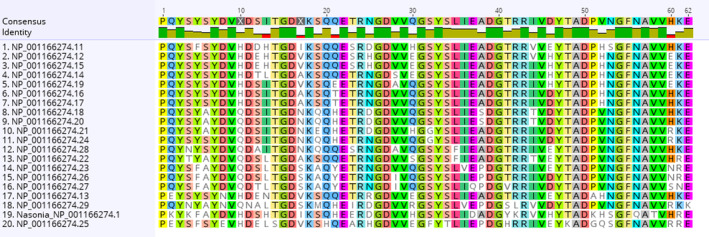
Unique *N. lecontei* Hisnavicin amino acid motif alignment. Residues identical to the consensus are colored.

As part of the innate immune system, antimicrobial peptides (AMPs) are expressed upon infection to kill or inhibit microbes. Based on hymenopteran sequences, the *N. lecontei* AMP gene family had 21 genes (Table 1), including single copies of *Hymenoptaecin*, *Abaecin*, and *Tachystatin*, but not a clear *Defensin* ortholog (Tables A6 and A9). Eighteen *Hisnavicin* genes were identified, including a *Neodiprion*‐specific expansion of 10 histidine‐rich paralogs orthologous to cuticle proteins in *Harpegnathos saltator* (ant) and to cuticular protein precursors in *Apis mellifera* (honeybee) (Figure A9). The *N. lecontei* Hisnavicins had a conserved 62 amino acid motif that appeared up to 19 times in a single protein (Figure A11); the purpose of this amplification is unknown. 95% of the AMPs were in genomic clusters (Figure 4). To date, this is the second largest AMP family documented in Hymenoptera: only *Nasonia vitripennis* has more AMPs than *N. lecontei* (44 vs. 21) (Tian et al., 2010). However, relatively few hymenopteran genomes have AMP annotations, making it difficult to draw firm conclusions about AMP family size variation across Hymenoptera (Table A9). Due to the low bootstrap support of many branches in our Hisnavicin protein tree, we could not identify unambiguous *Neodiprion*‐specific clades (Figure A9). However, our *Neodiprion* cDNA tree (Figure A10) had strong support for a monophyletic cluster of eight *Hisnavicins* that were part of a larger cluster on linkage group 5 (Figure 4); the eight gene cluster had some evidence of non‐neutral evolution; however, the 2‐ratio foreground branch *ω* value was less than the background value (Table 2).

**FIGURE A12:**
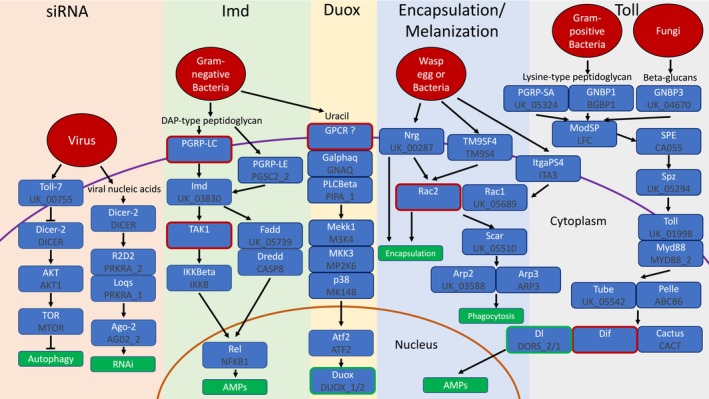
Innate immune pathways overview (based on *Drosophila melanogaster*).

Outside of the AMP family, most immune pathways had direct orthologs with *D. melanogaster* (Figure A12, Table A7). The basic viral siRNA response pathway was completely conserved between species. The immune deficiency (IMD) pathway was missing an ortholog for the peptidoglycan recognition receptor *PGRP‐LC*, but it is likely that another *PGRP* replaced *PGRP‐LC* in *N. lecontei*; assigning PGRP orthology was also difficult in ants (Gupta et al., 2015). Also missing is the *Drosophila* mitogen activated protein kinase kinase kinase, TGF‐β activated kinase 1 (*Tak1*), but *N. lecontei* had a similar TGF‐β activated kinase that is a close ortholog to several *Tak1‐like D. melanogaster* proteins possibly involved in immune deficiency signaling. The encapsulation/melanization pathway was missing one of the two *Drosophila* GTPases (*Rak2*); the *N. lecontei Rak1* ortholog may be playing both roles, but again this is likely due to the difficulty of assigning one‐to‐one orthologs. The Duox pathway was missing the top G‐protein coupled receptor, but this is unknown in *D. melanogaster* and unidentified in other Hymenoptera (Evans et al., 2006). Interestingly, *N. lecontei* had two copies of Dual Oxidase (*Duox*), which regulates commensal gut microbiota and infectious microbes (Ha et al., 2005; Lee et al., 2015); *Apis mellifera* had one copy. Finally, the Toll pathway *NF‐kappaB* transcription factor, *Dorsal‐related immunity factor (Dif)* does not have a one‐to‐one ortholog in *N. lecontei*, but two copies of its paralog, *Dorsal*, were present. The sawflies *Athalia rosae* and *Orussus abietinus* each had one *Dorsal* ortholog and no *Dif* orthologs (Oeyen et al., 2020). Since *Dif* is also missing in the Apocrita (Oeyen et al., 2020), *N. lecontei* may have a *Dorsal* duplicate not found in the other sawflies. However, *N. lecontei* also had single copies of *Toll‐1* and *spaetzle*; other Hymenoptera (including *A. rosae* and *O. abietinus*) have at least five copies of *Toll‐1* and two copies of *spaetzle* (Table A7). Since the Toll pathway regulates the expression of some AMPs (Lourenço et al., 2018; Zambon et al., 2005), it is possible that in *N. lecontei* the *Hisnavicin* gene expansion is compensating for the loss of *Toll‐1* and *spaetzle*.

### 2.3 Limitations of assembly and annotation for interspecific analyses

Comparative genomics is a powerful approach for evaluating the repeatability and predictability of evolutionary outcomes; however, the comparisons are only as good as the underlying data. The draft genome presented here was made solely from small‐insert libraries with Illumina HiSeq short reads; these types of assemblies are more fragmented with hundreds to thousands of scaffolds (Table A2). Fragmentation increases the possibility of missing or partial gene annotations since genes may be split across scaffolds, which was the case for the *N. lecontei* olfactory receptor genes NlOR46joi, NlOR54joi, NlOR56joi; missing gene annotations would affect our tests for positive selection and interpretations of genomic clustering. In fact, during the writing of this paper a new *N. lecontei* genome made with sequencing technologies that supplement short reads with long reads (Korlach et al., 2017) and reveals structural information (Peart et al., 2021) became available (Herrig et al., 2023). This provides a future opportunity to revisit these limitations and assess how assembly method and quality affects conclusions about gene family evolution.

Besides genome assembly continuity, gene annotations themselves may be difficult to compare directly. Across studies that included manual annotations, we observed a lack of consistency in the methods and criteria for manually curated gene family datasets. The most problematic inconsistencies were the criteria for delineating intact, partial, and pseudogenized gene annotations. “Intact” could mean an exon‐by‐exon check against closely related orthologs, a minimum amino acid length, or simply the presence of an expected domain. Furthermore, the number of pseudogenized and partial annotations were not always reported or were conflated. This is in addition to variation in the methods used to search for genes. Inconsistency in annotation methods and criteria across studies may introduce taxon‐specific biases in gene number unrelated to natural selection.

### 2.4 The role of gene family evolution in pine specialization

During a niche shift, novel selective pressures may favor the gain or loss of genes within environmentally responsive gene families. For example, pseudogenization and gene loss have been documented in diverse host‐specialized taxa and in multiple gene families (Cao et al., 2014; Goldman‐Huertas et al., 2015; McBride, 2007; Smadja et al., 2009; Suzuki et al., 2018). *Neodiprion lecontei* feeds on a single genus of host plants (*Pinus*) and is an exemplar of a family that shifted from angiosperms to coniferous host plants sometime in the last 60 million years (Peters et al., 2017). When this shift occurred, some genes important for adaptation to angiosperm hosts may have experienced relaxed selection or positive selection for loss‐of‐function mutations. A priori, one gene family for which we expected evidence of gene loss was the AMP family. Our rationale was that the presence of antimicrobial and fungicidal compounds in pine resin (Cowan, 1999; Gershenzon & Dudareva, 2007; Grayer & Harborne, 1994; Himejima et al., 1992) may have led to relaxed selection on genes involved in immunity. Moreover, because immunity is costly (Sheldon & Verhulst, 1996), selection may have favored a reduced innate immune response in pine feeders. In support of our logic, honeybees (*Apis mellifera*) exposed to plant resin have reduced expression of immune‐related genes (Simone et al., 2009) and wood ants (*Formica paralugubris*) that use conifer resin as building material have slightly reduced inducible immune system activity and nests with lower bacterial and fungal loads (Castella et al., 2008). In Diptera, AMP loss is associated with herbivorous lineages that live within host tissue, a more sterile habitat than is experienced by most dipterans (Hanson et al., 2019).

Consistent with our prediction that there would be AMP loss in *N. lecontei*, we were unable to find a clear ortholog of *Defensin*, an AMP gene present in all dipterans (Hanson et al., 2019) and almost all hymenopterans (Table A9) tested to date. However, the most striking pattern we observed in the AMP family in *Neodiprion lecontei* was a putative *expansion* of ~18 *Hisnavicin*‐like AMPs. One interpretation of this lineage‐specific proliferation of AMP genes is that a shift to conifers favored gene gain. Although we did not see evidence of positive selection among *Hisnavicin*‐like paralogs (Table 2), it remains possible that novel selection pressures associated with pines—perhaps a community of pathogens unique to pines—favored the retention of *Hisnavicin* duplicates because they produced a beneficial increase in gene dosage (e.g., Perry et al., 2007). However, additional data are needed to confirm that *Hisnavicin* orthologs act as AMPs in *N. lecontei* and to characterize AMPs in closely related symphytan families to verify that this expansion is unique to conifer‐feeding sawflies.

Outside of the AMPs, we detected pseudogenes in the OR, GR, and CYP families. However, the prevalence of pseudogenes was modest in comparison to other host‐specialized insect taxa. For example, in the *N. lecontei* chemosensory protein gene families (ORs, GRs, and OBPs)—the families most often associated with specialization‐associated gene loss (Goldman‐Huertas et al., 2015; Matsuo et al., 2007; McBride, 2007; McBride & Arguello, 2007)—we found that only 0%–5% of genes had clear evidence of a loss‐of‐function mutation. One explanation is that the pseudogenization events that accompanied adaptation to coniferous plants are no longer detectable in the *Neodiprion lecontei* genome. For example, in *Drosophila*, pseudogenes have an estimated half‐life of ~14.3 million years (Petrov et al., 1996; Petrov & Hartl, 1997, 1998). Alternatively, the prevalence of host‐associated pseudogenization may vary across insect lineages and gene families. Additional data—from both closely related sawfly lineages and diverse insect taxa—are needed to evaluate these explanations, which are not mutually exclusive.

A shift to pine‐feeding may have also favored gene gain, especially in gene families that mediate the detection of and response to pine‐specific volatiles and defenses. Of the gene families we examined, four had clusters of more recently diverged paralogs with common ancestry sometime after *Neodiprion* diverged from its closest symphytan relative (based on available gene annotations) (Table 1, Figures A1, A3, A7, A9). Many of these closely related paralogous groups are also clustered in the genome, suggesting they originated via tandem duplication (Figure 4). The largest putative expansion was the group of 15 *Hisnavicin*‐like AMPs discussed above. However, we also observed putative expansions in the OR, GR, and CYP families that showed evidence of positive selection (Table 2).

One *Neodiprion‐*specific GR clade with evidence of positive selection—GR clade 1—is an expansion of six paralogs (one is pseudogenized) orthologous to *DmG33a*, one of three co‐receptors required for caffeine detection (Lee et al., 2009; Moon et al., 2006, 2009). However, orthologs were not found for *DmGR93a* (Lee et al., 2009) and *DmGr66a* (Moon et al., 2006). Interestingly, in *Drosophila*, *DmGR93a* is a fine‐tuned receptor with a higher specificity for caffeine while *DmG33a* and *DmGr66a* are more broadly tuned and participate in the detection of other bitter compounds (Shim et al., 2015); bitter compounds are usually interpreted as a deterrent signal (Yarmolinsky et al., 2009). Nevertheless, honeybees, which also lack clear orthologs to these putative co‐receptors (Robertson & Wanner, 2006), can detect and even prefer low concentrations of caffeine and nicotine (Singaravelan et al., 2005 but see de Brito Sanchez, 2011). Although pines do not contain caffeine, they synthesize alkaloids that could confer bitterness (Mumm & Hilker, 2006). Thus, despite not having paralogous expansions for all the caffeine receptors, members of GR clade 1 may still be involved in the detection of pine‐specific bitter compounds. Duplications of putative bitter GRs are documented in other host‐specialized insects, such as *Heliconius*, *Danaus*, and *Bombyx* butterflies and other lepidoptera (Briscoe et al., 2013; Engsontia et al., 2015; Wanner & Robertson, 2008). Together, these observations lend support to the hypothesis that GR bitter receptors are frequently involved in plant‐feeding insect host shifts and host specialization.

Because pines contain toxic components like terpenoids and phenolics, detoxifying gene families are also promising candidates for pine adaptation. The mountain pine beetle (*Dendroctonus ponderosae*), feeds on pine bark and wood and has gene “blooms” (species‐specific paralogous gene expansions) in the CYP3 and CYP4 clans (Keeling et al., 2013). Similarly, the CYP family in *N. lecontei* had five blooms (Table 1, Figure A7): four CYP3 and one CYP4. CYP3 blooms are also found in wood‐feeding insects that do not use pine, such as the emerald ash borer (*Agrilus planipennis*) (David Nelson, unpublished data) and the Asian longhorned beetle (*Anoplophora glabripennis*) (McKenna et al., 2016). Notably, *N. lecontei* larvae frequently ingest pine bark in addition to pine needles (Wilson, 1991), suggesting that CYP3 may expand predictably in wood feeders. Additionally, one of the two *Neodiprion*‐specific CYP3 clades with possible evidence of positive selection (CYP clade 3) (Table 2) is from the CYP6 subfamily, linked to host plant adaptation in several insect taxa (Feyereisen, 2012; Li et al., 2003, 2007; Mittapelly et al., 2019).

## 3 SUMMARY AND CONCLUSIONS

The predictability of gene family expansion or contraction in response to specific selection pressures is still an open question. Here, we investigated the evolutionary history of five environmentally responsive gene families in the *N. lecontei* draft genome, a hymenopteran exemplar of a pine‐specialized lineage. Although we saw minimal evidence of recent gene loss via pseudogenization, at least four gene families (OR, GR, CYP, and AMP) had patterns consistent with recent expansions, and three of these families (OR, GR, and CYP) also had possible evidence of positive selection within *Neodiprion*‐specific clades. Based on these data, we hypothesize that these gene families contributed to pine adaptation in diprionids and possibly other host‐specialized insects. Testing this hypothesis requires the comparative analysis of high‐quality assembly and annotation data from phylogenetically and ecologically diverse insect species. For hymenopterans, increased effort in understudied symphytan, parasitoid, and herbivorous taxa would be especially useful for disentangling different axes of ecological variation contributing to changes in gene family size. For greater insight, annotation data from a greater diversity of environmentally responsive gene families—that is, families other than chemosensory genes—are also needed. To maximize signal: noise ratio across diverse taxa and genes, rigorously observed standardized protocols for annotation conventions are sorely needed (Klimke et al., 2011). Together, these data will make it possible to determine the extent to which certain gene families expand and contract predictably in response to ecology.

## 4 MATERIALS AND METHODS

### 4.1 Biological material

To minimize the confounding effects heterozygosity has on genome assembly, we sequenced haploid siblings. Like all Hymenoptera, sawflies have haplodiploid sex determination in which males (haploid genomes) emerge from unfertilized eggs and females (diploid genomes) from fertilized eggs. A virgin female will bear a clutch of all‐male offspring with haploid recombinants of the maternal genome. But the individual genomes are not identical, so an assembly derived from a single clutch is akin to a diploid assembly made from a single individual.

All insects were reared in custom, climate‐controlled environmental chambers (18:6 light cycle, 22°C, 70% RH) on jack pine (*Pinus banksiana*) foliage. Our laboratory line of *N. lecontei* was established from multiple larval colonies collected from a mugo pine (*P. mugo*) in Lexington, Kentucky, USA (37°59′01.6″ N 84°30′38.8″ W; population ID: RB017). For the transcriptome, adults and larvae were collected from the first laboratory‐reared generation; both were stored at −80°C. For the genome assembly, the founding population was propagated in the lab for two generations, followed by brother–sister matings for an additional two generations. At this point, a single, virgin, adult female (I2G2‐V, 4th generation in the lab) was allowed to lay unfertilized eggs onto jack pine seedlings. The offspring (haploid male brothers from an inbred mother) were reared until the eonymph (prepupal) life stage, at which point they were isolated without food for 24 h prior to preservation in absolute ethanol at −20°C. Although eonymphs are nonfeeding, they were starved to minimize residual gut content.

### 4.2 Sample preparation and sequencing

#### 4.2.1 Genomic DNA

Whole eonymph bodies were individually frozen inside microcentrifuge tubes with liquid nitrogen and ground with pestles made from 1‐mL micropipette tips; the resulting powder was incubated in cetyltrimethylammonium bromide (CTAB) buffer supplemented with proteinase K and RNase A. After phenol–chloroform–isoamyl alcohol (PCI) extraction and ethanol precipitation, the precipitate was dried overnight before being resuspended in tris‐EDTA (TE) buffer. DNA integrity was assessed with 0.7% agarose gel, purity was measured with the 260/280 ratio, and concentration was measured with a Quant‐iT dsDNA High‐Sensitivity fluorescence assay (Thermo Fisher Scientific).

The HudsonAlpha Genomic Services Lab (Huntsville, AL, USA) prepared and sequenced the DNA libraries. Two small‐insert, barcoded libraries with average fragment sizes of 337 bp and 864 bp were made from a single individual. A 4.6‐kbp mate‐pair, barcoded library was made from 25 pooled individuals. All individuals were brothers from the same I2G2‐V mother. The libraries were sequenced on Illumina HiSeq 2000 with paired‐end, 100 bp (PE100) reads: the small‐insert libraries each had ¼ of a flow cell lane and the mate‐pair library had an entire lane.

#### 4.2.2 mRNA

The RNeasy Mini extraction kit (Qiagen) was used to collect total RNA from adult female body, adult female head, adult male body, adult male head, eonymph body, feeding larval body, and feeding larval head. RNA from the eonymph head was extracted but not sequenced due to insufficient yield. Each tissue was represented with one replicate that had equal RNA contributions from eight individuals, except for the eonymph body which was comprised of three individuals. RNA integrity and concentration were measured with a 2100 Bioanalyzer (Agilent).

The HudsonAlpha Genomic Services Lab (Huntsville, AL, USA) handled library preparation and sequencing. Nonstranded, barcoded libraries were made for each of the seven tissue samples; on average, mRNA was sheared to 200 bp. The libraries were combined and sequenced on an entire flow cell of Illumina MiSeq with PE250 reads in addition to one lane of Illumina HiSeq 2000 with PE50 reads.

### 4.3 Read processing and assembly

#### 4.3.1 De novo genome assembly

Sequencing reads were chastity‐filtered and adaptor‐trimmed with fastq‐mcf (ea‐utils v1.04.803) (Aronesty, 2011), and quality‐filtered with fastq_quality_filter (FASTX Toolkit v0.0.13.2) (Gordon & Hannon, 2019). The 337‐bp small‐insert reads and the 4.6‐kbp mate‐pair reads were quality‐filtered to retain reads where at least 80% of the bases had a quality score of 20 or higher (parameters: ‐q 20 ‐p 80). Due to sequencing quality, the 864‐bp small‐insert reads were filtered to retain reads where at least 70% of the bases had a quality score of 20 or higher (R1) or 60% (R2) (parameters: ‐q 20 ‐p 60/70). In situations where only one end of the paired‐end reads passed filtering, the passed reads were kept and treated as single‐end data. Kmer counting was used to measure read depth before and after filtering (Jellyfish v1.1.11) (Marçais & Kingsford, 2011). Finally, reads were screened for sequencing contamination by mapping the reads (BWA v0.7.12‐r1039) (Li & Durbin, 2009) to reference genomes for *Escherichia coli* (K12 substr. DH10B uid58979), human (v37), loblolly pine (*Pinus taeda*, v0.8), and *Wolbachia* (endosymbiont of Dmel uid57851).

The genome was assembled with ALLPATHS‐LG (v47417) (Gnerre et al., 2011) using default settings, including a minimum scaffold size of 1000 bp. The error‐correction module was run on the reads prior to assembly. After assembly, GapFiller (v1.11) (Boetzer & Pirovano, 2012) was used to help close intrascaffold gaps. Spurious scaffolds were identified with SOAP. coverage (v2.7.7) (Li et al., 2009): reads were mapped to the assembly scaffolds and scaffolds with a read depth <15 and nucleotide percentage <40 were removed. The completeness of the final assembly was measured with CEGMA (v2.5) (Parra et al., 2007) and BUSCO (v1.22) (Simão et al., 2015) benchmarks. BUSCO was run with the arthropoda‐25oct16 database (parameters: –‐long).

#### 4.3.2 De novo transcriptome assembly

For both the PE250 MiSeq and the PE50 HiSeq reads, fastq‐mcf (ea‐utils v.1.04.803) (Aronesty, 2011) was used for chastity filtering and Trimmomatic (v0.32) (Bolger et al., 2014) was used to adaptor clip, trim, and quality‐filter. The PE250 MiSeq reads were processed with the Trimmomatic parameters ILLUMINACLIP: 2:15:5, HEADCROP: 10, CROP: 60, MINLEN: 60, AVGQUAL: 25 whereas the PE50 HiSeq reads were processed with ILLUMINACLIP: 2:15:5, HEADCROP: 15, MINLEN: 35, AVGQUAL: 25. Because the mRNA libraries had an average insert size of 200 bp, the MiSeq reads required extensive adaptor trimming. Reads were screened for contamination as described in “*De novo genome assembly”*.

For each tissue, transcriptomes were assembled with Trinity (r2013_08_14) (Grabherr et al., 2011; Haas et al., 2013) using default settings and the ‐‐jaccard_clip option. Spurious sequences were identified by mapping the sequencing reads to the assembled transcripts with RSEM (v1.2.18) (Li & Dewey, 2011); transcripts with either FPKM or TPM values <1 were removed. After filtering, the transcriptomes were combined, and duplicate sequences were removed.

### 4.4 Genome size estimation

Flow cytometry was described in (Harper et al., 2016). For genome size estimation, we used adult males and females from a lab line of *N. lecontei* established from a colony collected in Auburn, GA (33°59′22.4″ N, 83°47′44.6″ W; population ID: RB027). Briefly, cell nuclei were collected from the heads of 7 individuals (4 female, 3 male) and stained with propidium iodide. Mean fluorescence for each sample was measured with a BD FACSCalibur flow cytometer (BD Biosciences) and compared to two external standards: *Drosophila melanogaster* (adult female heads, 1C = 175 Mbp) and *Gallus gallus domesticus* (CEN singlets from BioSure, Grass Valley, CA, 1C = 1222.5 Mbp). To correct for ploidy differences between haploid males and diploid standards, we multiplied the *N. lecontei* male estimates by 2. To obtain a single size estimate for each *N. lecontei* sample, we averaged values obtained for the two standards.

### 4.5 Repeat annotation

The *N. lecontei* genome assembly was masked with a custom repeat library. A lineage‐specific de novo repeat library was made with RepeatModeler (v1.0.7) (Smit & Hubley, 2008–2015) and combined with the hymenopteran repetitive element database (Nov. 2013) from Repbase (Jurka et al., 2005). The custom library was used by RepeatMasker (v4.0.3) (Smit et al., 2013–2015) (parameters: ‐cutoff 250 ‐s ‐pa 15 ‐gc 40 ‐a –poly) to identify and mask repetitive elements in the genome, including low‐complexity DNA and simple repeats.

Transposable element (TE) family consensus sequences were identified by rerunning RepeatModeler (Smit & Hubley, 2008–2015) on the genome assembly using the “ncbi” search engine. The resulting sequences were provided to RepeatMasker (Smit et al., 2013–2015) as a custom library to locate associated TE copies in the genome (parameters: ‐gc 40 ‐cutoff 250 ‐gff ‐gccalc ‐norna ‐nolow ‐no_is –poly). TE families with at least 10 fragments longer than 100 bp were extracted for further analysis.

The sequencing reads were mapped to a concatenation of the masked genome and the consensus TE sequences (BWA MEM (parameters: ‐M; Li & Durbin, 2009). Families that had at least 1× the median coverage to the reference genome for at least 80% of their sequence (to support at least one full insertion found by RepeatModeler) and at least 2x the maximum coverage of the reference genome (to support multiple insertions of the family) were extracted with genomeCoverageBed (BEDtools; Quinlan & Hall, 2010). We attempted to identify the consensus sequences with BLASTN and BLASTX (Altschul et al., 1990) searches against a database of repeat elements, but the only hits were to the lineage‐specific elements identified by RepeatModeler. Sequences were also filtered for BLAST hits to rRNA or mitochondrial sequences.

We also used dnaPipeTE (Goubert et al., 2015) to identify what proportion of our short reads was composed of repetitive content, we used a random subset of reads corresponding to 1‐fold coverage of the genome (331 Mb) and took the total for three separate random samplings of reads (parameters: genome size = 331,000,000 genome coverage = 1 samples number = 3). We then compared this annotation to the RepeatModeler annotation.

### 4.6 Gene and functional annotation

#### 4.6.1 Automated gene annotation

RNA‐Seq data for *N. lecontei* was used to generate training models for gene prediction along with utilization of peptide sequences from other species. PASA (r20130425beta) was used to build a comprehensive transcriptome set from Trinity assembled transcripts along with RNA‐Seq read mapping predictions generated from the Tuxedo pipeline. To improve annotation quality, in addition to this *N. lecontei* transcriptome, annotated proteins from *Atta cephalotes* (OGSv1.2), *Acromyrmex echinatior* (OGSv3.8), *Apis mellifera* (OGSv3.2), *Athalia rosae* (OGSv1.0), and *Nasonia vitripennis* (OGSv1.0) were provided to Maker (2.09) (Cantarel et al., 2008) as evidence for structural gene prediction. Prior to annotation, the genome was masked using a custom repeat database built using RepeatModeler (v1.0.8) and the annotation was run using the ab initio gene predictors Augustus, Genemark‐ES and snap in addition to the evidence provided. The functions of the predicted protein‐coding genes were putatively established with BLASTP alignments (Altschul et al., 1990) to the Swiss‐Prot database (accessed 20 April 12) (Apweiler et al., 2004). In cases of multiple matches, the top‐ranked alignment was assigned to the gene annotation. Protein motifs and functional domains within the annotations were also identified with an InterProScan (v5.3.46.0) (Jones et al., 2014) search against the InterPro database with gene ontology and IPR lookup (Finn et al., 2016). For the official gene set (OGS), the Maker annotations were filtered by hits to the reference databases and/or a minimum eAED score of 0.1. A second set of gene annotations was generated with the NCBI GNOMON pipeline (annotation release 100 on Nlec1.0 assembly, GCF_001263575.1) (Souvorov et al., 2010).

As the genome was annotated prior to submission to NCBI, we encountered a problem when the NCBI contamination software flagged vector/adaptor sequences for removal; this would disrupt the coordinates provided by Maker. We used a modified version of GAG (Geib et al., 2018) that could accept the flagged coordinates from NCBI to edit the assembly and update annotation coordinates accordingly.

#### 4.6.2 Chemoreceptor genes

The olfactory (OR) and gustatory (GR) receptor genes were manually curated following Robertson et al. (2003) and Robertson and Wanner, 2006). Amino acid sequences of manually curated chemoreceptor genes from *Apis mellifera* (Robertson & Wanner, 2006; Smith, Smith, et al., 2011), *Bombus terrestris* (Sadd et al., 2015) and *Cephus cinctus* (Robertson et al., 2018), *Drosophila melanogaster* (Flybase release FB2017_04), and *Nasonia vitripennis* (Robertson et al., 2010) were used as queries in TBLASTN (v2.2.19) (Altschul et al., 1990) searches against the *N. lecontei* draft genome (parameters: ‐e 100,000 ‐F F). Gene models were manually built in TextWrangler (v5.5) (Bare Bones Software), using protein alignment to identify exons and refine the gene structures; alignments were visualized with Clustal X (v2.1) (Larkin et al., 2007). The Neural Network Splice Predictor program from the Berkeley *Drosophila* Genome Project was used to help identify intron splice sites (http://www.fruitfly.org/seq_tools/splice.html). New gene models were added to TBLASTN searches and this process continued iteratively until new chemoreceptors were no longer found. The gene models were checked against RNA‐Seq reads from tissue‐specific transcriptomes (adult antennae, mouthparts, heads, legs, genitalia, and larval heads; Herrig et al., 2021) and against orthologs in the *N. pinetum* draft genome assembly (NCBI accession GCA_004916985.1).

#### 4.6.3 Odorant‐binding proteins

Custom scripts were used to identify Maker gene annotations (see Section 4.6.1) that contained the classic/6C, Plus‐C, Minus‐C, or atypical odorant‐binding protein (OBP) motif (Xu et al., 2009). These as well as OBPs from *Apis mellifera* and *Nasonia vitripennis* were used as queries for TBLASTN (Altschul et al., 1990) searches against the *N. lecontei* genome; searches did not yield any new OBPs. All genomic regions identified as potential OBPs were manually curated as described for chemoreceptor genes. After manual annotation, duplicate annotations or genes that lacked OBP motifs were removed.

#### 4.6.4 Cytochrome P450 genes

A broad set of 52 insect CYP genes (covering the diversity of insect CYP families) was searched against the *N. lecontei* genome assembly (*E*‐value cutoff 1e3). Scaffolds with hits were then searched against 8782 known insect CYPs. The top 10 hits were returned (later increased to 15 to recover more sequences) and filtered for duplicates. An alternative search of the NCBI GNOMON predictions (“Neodiprion lecontei[orgn] AND P450 NOT reductase”) was also performed and new sequences were added to the dataset. This approach found all the loci identified by the initial search, indicating that the GNOMON annotation tool was able to comprehensively search for CYP sequences. Finally, the candidate *N. lecontei* CYP sequences were manually curated based on comparison to the best BLAST hits.

#### 4.6.5 Immune‐related genes

Because of the relative completeness of its immune annotation, *Drosophila melanogaster* immunity genes were used to guide annotation. Reference immune genes from *D. melanogaster* tagged with the gene ontology term “GO:0002376 – Immune system process” were compiled from Flybase (release 6.13). Orthology with *N. lecontei* proteins was assigned initially with reciprocal BLASTP (Altschul et al., 1990) searches (*E*‐value cutoff 1e‐10). Reference *D. melanogaster* genes without obvious one‐to‐one orthologs in *N. lecontei* were examined individually to determine whether closely related paralogs in one or both species interfered with the inference of orthology. If not, they were searched against the *N. lecontei* genome assembly using TBLASTN (Altschul et al., 1990) in an attempt to identify unannotated orthologs.

Since antimicrobial peptides (AMP) are unlikely to be conserved between *D. melanogaster* and *N. lecontei*, AMPs from three representative hymenopterans *Apis mellifera* (Danihlík et al., 2015), *Nasonia vitripennis* (Tian et al., 2010), and *Camponotus floridanus* (Gupta et al., 2015; Ratzka et al., 2012; Zhang & Zhu, 2012) were used for BLAST queries. Furthermore, since AMP copy number is fast evolving, we attempted to find all the *N. lecontei* orthologs of each hymenopteran AMP instead of focusing on one‐to‐one orthology. Once again, BLASTP searches were performed against the annotated proteins and TBLASTN searches were performed against the assembled genome; the TBLASTN search did not reveal additional AMPs. Putative *N. lecontei* orthologs were reciprocally blasted against the appropriate hymenopteran proteome to assure that the best hits were indeed AMPs.

Amino acid and cDNA sequences for all manual annotated genes are available at Dryad. https://doi.org/10.5061/dryad.n8pk0p320

### 4.7 Glomeruli counts

#### 4.7.1 Antennal lobe histology

Whole heads of adult *N. lecontei* of both sexes were fixed in 2% paraformaldehyde, 2% glutaraldehyde in phosphate‐buffered saline (PBS) for 5 days. Heads were rinsed for 40 minutes three times and the brains dissected out in cold PBS. Following blocking with goat serum, brains were permeabilized with 1% Triton X‐100 in PBS (Electron Microscopy Supply, Fort Washington, PA; PBS‐TX), rinsed with 0.1% PBS‐TX, and incubated on a shaker at 25°C for three nights in primary antibody (1:500 in 2% goat serum in 0.2% PBS‐TX). Monoclonal *Drosophila* synapsin I antibody (SYNORF1, AB_2315426) from the Developmental Studies Hybridoma Bank (catalog 3C11) was used to label synapsin. Subsequently, brains were washed in 0.1% PBS‐TX and incubated for two nights in Alexa Fluor 568 (Thermo Fisher) goat antimouse secondary antibody (1:100 in PBS) in the dark at room temperature on a shaker. After secondary incubation, brains were rinsed with distilled water, dehydrated in increasing concentrations of ethanol, and mounted in custom‐made aluminum well slides. Brains were cleared by removing ethanol and replacing it with methyl salicylate. Brains were imaged on an inverted Zeiss 880 Laser Scanning Confocal Microscope with a 20X plan‐Apochromat 20x 0.8 aperture objective and optically sectioned in the horizontal plane at 3‐micron intervals.

#### 4.7.2 Glomeruli segmentation

Whole‐brain images of one female and one male were manually segmented using the TrakEM2 software package in ImageJ (Cardona et al., 2012; Schindelin et al., 2012). Individual glomeruli were traced in both brain hemispheres. Glomeruli near the center of the antennal lobe can be difficult to distinguish, meaning counts are biased toward fewer glomeruli and the largest number of glomeruli confidently detected represents a minimum of the number of expected glomeruli. Male *Neodiprion* have a collection of smaller synaptic clusters in their antennal lobe (Dacks & Nighorn, 2011), but the functional significance of this anatomy is not known. There are more than 50 of these smaller synaptic clusters and we suspect they do not represent the traditional one‐to‐one OR‐to‐glomerulus organization. Therefore, these structures were not included in counts. Male glomeruli number may be lower if particular OSNs contribute to these clusters instead of forming traditional glomeruli.

#### 4.7.3 Clustering and pseudogene analyses

To evaluate the extent to which members of our five focal gene families were located in tandem arrays, we placed our annotated genes on a linkage‐map anchored version of the *N. lecontei* genome assembly described in (Linnen et al., 2018). We considered genes to be clustered if they were located within a genomic region of 20(*n* – 1) kilobases, where *n* is the number of genes in the cluster under consideration. This criterion was chosen based on average gene densities in *Nasonia* (Niehuis et al., 2010) and clustering criteria described *Drosophila* (Vieira et al., 2007). For scaffolds that could not be placed on linkage groups, we evaluated clustering only if genes were more than 20 kb from either scaffold end.

#### 4.7.4 Identification of Neodiprion‐specific clades and tests of positive selection

First, we identified clades unique to *N. lecontei*. For each gene family, a multispecies, amino acid phylogeny was constructed with manually curated annotations from *N. lecontei*, select Hymenoptera, and *D. melanogaster*. Intact sequences were size filtered (350 ≥ for GR, OR, CYP; 100 ≥ for histnavicin and OBP); pseudogenes and partial annotations were excluded. MAFFT alignments (v7.305b) (Katoh et al., 2002) (parameters: ‐‐maxiterate 1000 –localpair) were visually inspected to remove sequences with large alignment gaps, and sites with more than 20% gaps were removed with trimAl (v1.4.rev15 build[2013‐12‐17]) (Capella‐Gutiérrez et al., 2009) (parameters: ‐gapthreshold 0.8). Maximum likelihood phylogenies were made in RAxML (v8.2.4) (Stamatakis, 2014) (parameters: ‐f a ‐x 12,345 ‐p 12345 ‐# autoMRE) using protein substitution models chosen from ProtTest3 (v3.4.2) (Abascal et al., 2010; Darriba et al., 2011).

*Neodiprion*‐specific clades were defined as those with at least four *N. lecontei* genes (not including partial and pseudogenes) and reasonable bootstrap support (>70%). Second, the clades were confirmed with cDNA phylogenies for each *N. lecontei* gene family. Amino acid sequences were aligned as above, however, after alignment TranslatorX (Abascal et al., 2010) was used to map cDNA sequences to the amino acid alignment. After trimming, the cDNA alignments were passed to RAxML to construct maximum likelihood gene family trees with the nucleotide substitution model ‐m GTRGAMMA.

Site tests were conducted with codeml (part of the PAML package (PAML v4.9e; Yang, 2007) using the cDNA phylogenies and sequences as inputs. For each *Neodiprion*‐specific clade, the gene family cDNA phylogeny was pruned to remove all branches except for that clade. Codeml models M7, M8, and M8a were fitted to the cDNA sequence and phylogeny data. Likelihood‐ratio tests (chi‐square distribution = upper tail) were performed for the nested models M7‐M8 (null model M7 equally distributes amino acid sites across 10 classes of *ω* parameter values (*p*, *q*) against alternative model M8 that has an 11th class for positively selected sites) and M8‐M8a (null model M8a that has 11 classes and does not allow positive selection against alternative model M8).

For branch tests, the cDNA phylogenies for each *N. lecontei* gene family were used to compare the lineage‐specific clade to the rest of the gene family. To determine if the foreground branch dN/dS (i.e., the branch with the species‐specific expansion) was significantly higher than the background (i.e., the rest of the gene family), for that clade we ran a two‐ratio codeml model (Model = 2, fix_omega = 0) and a one‐ratio model (Model = 0, fix_omega = 0) and performed a likelihood‐ratio test (chi‐square distribution = upper tail). To determine if the foreground branch is evolving under selection (dN/dS ≠ 1), we performed a likelihood‐ratio test (chi‐square distribution = two tail) comparing the two‐ratio model to a two‐ratio neutral model (Model = 2, fix_omega = 1). Our rationale for using a two‐tailed test for neutrality and a one‐tailed test for comparing the foreground branch to the rest of the tree is that this would enable us to detect scenarios in which a locus evolving under purifying selection (dN/dS ≠ 1) experiences increased positive selection (or relaxed purifying selection) at some sites upon entry into a novel niche.

## AUTHOR CONTRIBUTIONS

**Kim L. Vertacnik:** Conceptualization (lead); data curation (lead); formal analysis (lead); investigation (lead); writing – original draft (lead); writing – review and editing (lead). **Danielle K. Herrig:** Formal analysis (equal); writing – original draft (equal). **R. Keating Godfrey:** Formal analysis (equal); writing – original draft (equal). **Tom Hill:** Formal analysis (equal); writing – original draft (equal). **Scott M. Geib:** Formal analysis (equal); writing – original draft (equal). **Robert L. Unckless:** Formal analysis (equal); writing – original draft (equal). **David R. Nelson:** Formal analysis (equal); writing – original draft (equal). **Catherine R. Linnen:** Conceptualization (lead); formal analysis (lead); supervision (lead); writing – original draft (lead); writing – review and editing (lead).

## FUNDING INFORMATION

United States Department of Agriculture National Institute of Food and Agriculture [2016‐67014‐2475 to CRL]; the Kentucky Science and Engineering Foundation [KSEF‐3492‐RDE‐019 to CRL]; the University of Kentucky [Lyman T. Johnson Fellowship to KLV].

## CONFLICT OF INTEREST STATEMENT

None declared.

## Data Availability

The genome assembly, official gene set (OGS), and transcriptome described in this paper (v1 versions) can be found at https://i5k.nal.usda.gov/neodiprion‐lecontei. On GenBank (NCBI), the genome assembly is labeled whole genome shotgun sequencing project accession PRJNA28045 and the genomic sequencing reads are RefSeq accession PRJNA312506. The transcriptome is transcriptome shotgun assembly accession GEDM00000000; this is a combined transcriptome of all seven tissue types. The mRNA sequencing reads for each tissue type was submitted separately under BioSample and short read archive accessions SAMN04302192 (adult female head), SAMN04302193 (adult female body), SAMN04302194 (adult male head), SAMN04302195 (adult male body), SAMN04302196 (feeding larval head), SAMN04302197 (feeding larval body), and SAMN04302198 (eonymph body). The predicted gene annotations on NCBI are from Gnomon, the NCBI annotation pipeline, and were not described in this paper. Finally, the clustering analysis was based on a linkage‐map anchored version of the genome assembly described in Linnen et al., 2018. This anchored assembly is denoted as v1.1 in NCBI and the *N. lecontei* i5k Workspace@NAL (USDA). The *N. lecontei* manually annotated gene sequences, alignment files for the Hymenoptera‐wide gene family phylogenies, and spreadsheet versions of the Appendix tables are available at Dryad (https://doi.org/10.5061/dryad.n8pk0p320).

## APPENDIX 2

**TABLE A1:** Sequencing libraries and read counts for the *N. lecontei* genome.

	Raw reads (PE)	Raw read depth	Filtered reads (PE)	Filtered read depth
337 bp small‐insert	156,465,022	85×	128,902,479	70×
864 bp small‐insert	81,874,705	35×	49,953,962	17×
4.6 kbp mate‐pair	110,199,710	28×	88,796,403	25×

**TABLE A2:** Comparison of genome assembly and annotation statistics from published hymenopteran draft genomes.

Assembly								
Species^a^	Genome size (Mbp)^b^	Length (Mbp)^c^	Total scaffolds	ScaffoldN50 (Mbp)	Total genes in OGS	GC content (%)	Repetitive sequence (%)	References
Sawflies								
*Athalia rosae*	170 (k)	164	522	1.37	11,894	41	5	Oeyen et al. (2020)
*Cephus cinctus*		162	1976	0.62	13,824	40	13	Robertson et al. (2018)
*Eupontania aestiva*	288 (k)	222	16,952	0.05	24,979	40	17	Michell et al. (2021)
*Euura lappo*	248 (k)	260	2503	0.21	23,848	41	27	Michell et al. (2021)
*Neodiprion lecontei*	331 ± 10 (fc)	239	4523	0.24	12,980	40	16	This paper
*Orussus abietinus*	247 (k)	201	936	2.37	10,959	45	17	Oeyen et al. (2020)
*Tenthredo notha (Klug)*		253	26	14.00	10,235			Falk et al. (2022)
Bees								
*Apis cerana*	239 (?)	228	2430	1.40	10,651	30	6	Park et al. (2015)
*Apis dorsata*		224		0.04	13,517	32	7	Oppenheim et al. (2020)
*Apis dorsata*		230	4040	0.73	12,172	33	18	Fouks et al. (2021)
*Apis florea*		229	7945	2.86	14,393	35	14	Fouks et al. 20 Updates Karpe et al. (2016)
*Apis laboriosa*		226	4376	3.34	11,466	32	7	Lin et al. (2021)
*Apis mellifera*	262 ± 1 (fc)	224	177	13.62	15,314	33	8	Amel_4.5, OG Wallberg et al. (2019). (update of Elsik et al. (2014))
*Bombus bifarius*		267	1249	2.20	13,325	38		Heraghty et al. (2020)
*Bombus breviceps*		246		4.04	16,904		17	Sun et al. (2021)
*Bombus confusus*		239		3.26	14,027		13	Sun et al. (2021)
*Bombus consobrinus*		249		4.77	15,570		16	Sun et al. (2021)
*Bombus cullumanus*		247		4.58	16,299		16	Sun et al. (2021)
*Bombus difficillimus*		248		2.07	14,303		15	Sun et al. (2021)
*Bombus haemorrhoidalis*		240		4.74	15,689		13	Sun et al. (2021)
*Bombus ignitus*		241		3.02	16,181		15	Sun et al. (2021)
*Bombus impatiens*		247	1505	1.40	15,896	38	18	Sun et al. (2021)
*Bombus opulentus*		242		2.42	16,181		15	Sun et al. (2021)
*Bombus picipes*		254		5.88	16,920		18	Sun et al. (2021)
*Bombus polaris*		246		2.25	15,575		16	Sun et al. (2021)
*Bombus pyrosoma*		253		6.07	16,432		15	Sun et al. (2021)
*Bombus sibiricus*		262		3.14	16,628		18	Sun et al. (2021)
*Bombus skorikovi*		242		4.34	16,308		16	Sun et al. (2021)
*Bombus soroeensis*		244		2.12	14,545		15	Sun et al. (2021)
*Bombus superbus*		230		6.90	15,261		10	Sun et al. (2021)
*Bombus terrestris*	433 (fc)	249	5678	3.50	11,875	39	15	Sadd et al. (2015)
*Bombus turneri*		243		4.34	16,970		15	Sun et al. (2021)
*Bombus vancouverensis*		282	1162	3.06	13,687	38		Heraghty et al. (2020)
*Bombus vosnesenskii*		276	1429	2.83	13,527	38		Heraghty et al. (2020)
*Bombus waltoni*		231		4.66	15,460		10	Sun et al. (2021)
*Ceratina calcarata*	364 (k)	262	3577	0.07	11,310	42		Rehan et al. (2016)
*Colletes gigas*	322 (k)	273	326	8.11	11,016	40	26	Zhou et al. (2020)
*Dufourea novaeangliae*	2 (fc); 291 (k)	291	84,187	2.40	12,453	40	37	Kapheim et al. (2015)
*Eufriesea mexicana*	(fc); 1000 (k)	557	3,522,543	0.00	12,022	41	49	Kapheim et al. (2015)
*Euglossa dilemma*	3200 (k)	588	22,698	0.14	15,904	40	39	Brand et al. (2017)
*Habropoda laboriosa*	377 (k)	294	650,185	1.30	13,279	39	26	Kapheim et al. (2015)
*Lasioglossum albipes*	416 (k)	342	41,433	0.62	13,448	42	33	Kocher et al. (2013)
*Megachile rotundata*	273 (k)	273	6266	1.70	12,770	37	43	Kapheim et al. (2015)
*Melipona quadrifasciata*	257 (k)	257	7386	1.90	15,368	39	18	Kapheim et al. (2015)
*Nomia melanderi*		300	3194	2.05	10,847	41	14	Kapheim et al. (2019)
*Osmia bicornis bicornis*	186 (k)	213	10,223	0.60	14,858	40	20	Beadle et al. (2019)
Ants								
*Acromyrmex echinatior*	335 (fc)	300	16,221	1.10	17,278	34	28	Nygaard et al. (2011)
*Atta cephalotes*	300 ± 1 (fc)	318	2835	5.20	18,093	33	25	Tsutsui et al. (2008)
*Atta columbica*	280 (k)	293		2.00	14,345	30	32	Nygaard et al. (2016)
*Camponotus floridanus*	238 (qPCR)	238	25,494	0.60	17,064	34	15	Cflo_v4 Bonasio et al. (2010)
*Cardiocondyla obscurior*		188	1854	3.10	17,552	40	21	Schrader et al. (2017)
*Cerapachys (Ooceraea) biroi*		214	4579	1.30	17,263	42	14	Cbir_v3; OGSv Oxley et al. (2014)
*Copidosoma floridanum*	557 (fd)	554		1.21	19,869	36		Thomas et al. (2020)
*Cyphomyrmex costatus*	319 (k)	323		1.02	16,468	30	34	Nygaard et al. (2016)
*Dinoponera quadriceps*		268	719	1.36	13,688	40	6	Patalano et al. (2015)
*Formica exsecta*		278	14,617	1.00	13,767	36	16	Dhaygude et al. (2019)
*Harpegnathos saltator*	297 (qPCR)	297	21,347	0.60	18,564	45	27	Hsal_v3 Bonasio et al. (2010)
*Linepithema humile*	251 ± 2 (fc)	220	3030	1.40	16,123	38	31	Tsutsui et al. (2008)
*Monomorium pharaonis*		284		0.02				Mikheyev and Linksvayer (2015)
*Pogonomyrmex anergismus*			3743		16,968	34		Smith et al. (2015)
*Pogonomyrmex barbatus*		235	4646	0.79	17,177	37	9–18	Smith, Smith, et al. (2011)
*Pogonomyrmex colei*			3467		16,910	34		Smith et al. (2015)
*Pseudomyrmex gracilis*	392 (k)	282	6556	0.35	16,069	39	40	Rubin and Moreau (2016)
*Pogonomyrmex rugosus*			3861		17,093	34		Smith et al. (2015)
*Solenopsis invicta* rk);	753.3 (fc)	353	10,543	0.72	16,569	36		Li and Heinz (2000)
*Trachymyrmex cornetzi*	396 (k)	402		0.63	19,827	30	42	Nygaard et al. (2016)
*Trachymyrmex septentrion*	294 (k)	295		2.45	15,575	30	24	Nygaard et al. (2016)
*Trachymyrmex zeteki*	295 (k)	269		1.33	15,530	30	25	Nygaard et al. (2016)
*Vollenhovia emery*		269	46,989		26,902	42		Smith et al. (2015)
*Vollenhovia nipponica*			40,233		25,102	30		Smith et al. (2015)
Wasps								
*Ampulex compressa*		279	18,453	9.13				Obiero et al. (2021)
*Ceratosolen solmsi*	294 (k)	278	7397	9.60	11,412	30	9	Xiao et al. (2013)
*Cerceris arenaria*		358	182,826	2.09				Obiero et al. (2021)
*Cotesia congregata*		207		1.12	14,140	28		Gauthier et al. (2021)
*Cotesia flavipes*		155	18,696	(contig N50)	17,381	30		Gauthier et al. (2021)
*Cotesia glomerata*		243	50,739	(contig N50)	23,498	29		Gauthier et al. (2021)
*Cotesia rubecula*		216	35,383	(contig N50)	22,795	29		Gauthier et al. (2021)
*Cotesia sesamiae*		166	13,504	(contig N50)	17,785	29		Gauthier et al. (2021)
*Cotesia vestalis* 203 (k)		178	1437	2.61	11,278	30	24	Shi et al. (2019)
*Cotesia vestalis*		176	31,915	(contig N50)	19,239	29		Gauthier et al. (2021)
*Diachasma alloeum*		389	3968	0.65	14,480	38	49	Tvedte et al. (2019)
*Diadromus collaris*	408 (k)	399	2731	1.03	15,328	37	37	Shi et al. (2019)
*Fopius arisanus*		154	1042	0.98	11,661	39		Geib et al. (2017)
*Gonatopus flavifemur*	1 (fc); 603 (k)	637	487	35.00	23,100	36	61	Yang et al. (2021)
*Leptopilina heterotoma*		463	83,487	0.00	15,576	28		Wey et al. (2020)
*Microplitis demolitor*	241 (fc)	241	1794	1.10	12,670	33		Burke et al. (2018)
*Nasonia vitripennis*	333 (fd)	295	6181	0.71	17,279	41	17	Nvit_2.1; nvi Werren et al. (2010)
*Nasonia vitripennis*		297	444	6.60				Dalla Benetta et al. (2020)
*Polistes canadensis*		211	3852	0.54	15,799	29	12	Patalano et al. (2015)
*Polistes dominula*	1 (fc); 246 (k)	208	1483	1.63	11,819	31	14	Standage et al. (2016)
*Trichogramma pretiosum*	199 (k)	195	357	3.71	12,928	40	30	Lindsey et al. (2018)
*Macrocentrus cingulum*	135 (k)	132	5696	0.19	11,993	36	22	Yin et al. (2018)

^a^ Assembly and official gene set (OGS) version are v1.0 unless reported otherwise.

^b^ Methods used to measure genome size: fc , flow cytometry; k , kmer analysis; fd , Feulgen densitometry; rk , reassociation kinetics; ? , unknown.

^c^ Scaffold length with gaps.

**TABLE A3:** Summary of elements identified in the *N. lecontei* genome.

	dnaPipeTE number of families	dnaPipeTE percentage	RepeatModeler number of families	RepeatModeler percentage (fraction of assembly)	RepeatModeler percentage (fraction of total genome)	Portion of repetitive genome missing in assembly percentage
TE Order						
LTR	64	3.2605	29	0.8601	0.6228	2.6377
LINE	62	0.9526	22	0.6415	0.4645	0.4881
SINE	29	0.0031	20	0.0042	0.003	0
DNA	63	3.8394	38	2.0358	1.4741	2.3653
MITE	0	0	0	0	0	0
Helitron	51	0.1384	32	0.1248	0.0903	0.0481
Repeat Order						
rRNA	21	0.7173	16	0.0173	0.0125	0.7048
Low complexity	8	0.6899	8	0.383	0.2773	0.4126
Satellite	37	1.922	21	0.3126	0.2263	1.6956
Tandem repeats	0	0	0	0	0	0
Simple repeat	8	3.7301	26	1.9386	1.4037	2.3264
Other						
Unknown	24	12.3394	122	9.5414	6.9089	5.4305
Total	367	27.5928	334	15.8593	11.4837	16.1091

**TABLE A4:** Tissue types, read counts, and transcript counts for the *N. lecontei* transcriptome.

	Raw MiSeq (PE250)	Raw HiSeq (PE50)	Filtered reads (combined)	Raw transcripts	Final transcripts
Adult female body	6,915,054	34,695,138	32,163,481	26,527	26,335
Adult female head	2,577,645	34,479,620	31,528,864	28,577	28,386
Adult male body	2,580,520	37,763,308	34,496,435	25,754	25,644
Adult male head	3,181,430	29,909,654	27,442,849	30,884	30,775
Feeding larval body	1,624,852	32,351,773	29,142,629	22,139	22,092
Feeding larval head	3,798,521	34,720,778	31,521,407	28,253	28,114
Eonymph body^a^	2,523,305	29,154,633	26,325,699	19,949	19,916

^a^ Due to a sample preparation error eonymph head mRNA was unavailable.

**TABLE A5:** Glomeruli counts from left and right antennal lobes of an adult *N. lecontei* male and an adult *N. lecontei* female.

Sex	Side	Glomeruli
Male	Left	37
	Right	37
Female	Left	49
	Right	45

**TABLE A6:** Summary of *N. lecontei* AMP orthology with other Hymenoptera.

AMP family	*Neodiprion lecontei* orthologs	*Nasonia vitripennis* orthologs	*Apis mellifera* orthologs	*Camponontus floridanus* orthologs
Hymenoptaecins	Nlec_unknown_03748‐mRNA‐1	Nahymenoptaecin‐1^a^ (NP_001165829.1 Nahymenoptaecin‐2 (NP_001234886.1)	Hymenoptaecin (NP_001011613.1)^b^	Hymenoptaecin (XP_019883221.1)
Abaecins	Nlec_unknown_03749‐mRNA‐1		Abaecin (NP_001011617.1)	
Tachystatin‐type	Nlec_unknown_04433‐mRNA‐3	Naickin‐1 (XP_016840398.1)		Cafickin1‐1 (XP_011257508.1)
Defensins	Nlec_TEN2_1‐mRNA‐1	Nasonin‐2 (NP_001171933.1)^c^		
Histidine‐rich	Nlec_CU21‐mRNA‐1 Nlec_PROML‐mRNA‐1	Hisnavicin‐4 (NP_001166274.1) Hisnavicin‐3 (NP_001166274.1)^d^		

^a^ Reciprocal best Blast hit when more than one sequence is listed.

^b^ Best blast hit for *A. mellifera* AMPs vs. *N. lecontei* proteins (not reciprocal).

^c^ Note that while the best blast hit for nasonin‐2 was Nlec_TEN2_1‐mRNA‐1, this was not a reciprocal best blast hit and Nlec_TEN2_1‐mRNA‐1 appears to be a teneurin.

^d^ Best blast hit for *N. vitripennis* AMPs vs. *N. lecontei* proteins (not reciprocal).

**TABLE A7:** Immunity gene orthologs between *N. lecontei* and *D. melanogaster*.

	Pathway	Gene	FBgn	Nlec ID	Orthology	%ID
Viruses	siRNA	Toll‐7	FBgn0034476	Nlec_unknown_00755‐mRNA‐1	RBH	53.1
		PI3Kp2E	FBgn0015279	Nlec_PK3CB‐mRNA‐1	RBH	45.5
		Akt1	FBgn0010379	Nlec_AKT1‐mRNA‐1	RBH	72.4
		Tor	FBgn0021796	Nlec_MTOR‐mRNA‐1	RBH	61.1
		Dicer‐2	FBgn0034246	Nlec_DICER_mRNA‐2	RBH	29.8
		Loqs	FBgn0032515	Nlec_PRKRA_1‐mRNA‐1	RBH	55.8
		R2D2	FBgn0031951	Nlec_PRKRA_2‐mRNA‐1	Dmel_v_Nlec	28
		Ago2	FBgn0087035	Nlec_AGO2_2‐mRNA‐1	Dmel_v_Nlec	37.1
		Drosha	FBgn0026722	Nlec_unknown_02096‐mRNA‐1	RBH	70.2
				Nlec_DUOX_2‐mRNA‐1		72.8
Gram‐negative	Duox	Duox	FBgn0283531	Nlec_DUOX_1‐mRNA‐1	RBH	36.4
		Galphaq	FBgn0004435	Nlec_GNAQ‐mRNA‐1	RBH	74.2
		PLCBeta	FBgn0262738	Nlec_PIPA_1‐mRNA‐1	RBH	65.9
		Mekk1	FBgn0024329	Nlec_M3K4‐mRNA‐2	RBH	46.7
		MKK3	FBgn0261524	Nlec_MP2K6‐mRNA‐1	RBH	65.7
		p38b	FBgn0024846	Nlec_MK14B‐mRNA‐1	RBH	79.1
		Atf2	FBgn0265193	Nlec_ATF2‐mRNA‐1	RBH	46.7
	Imd	PGRP‐LC	FBgn0035976	NA	NA	NA
		PGRP‐LF	FBgn0035977	Nlec_PGRP‐mRNA‐2	RBH	40.9
		PGRP‐LE	FBgn0030695	Nlec_PGSC2_2‐mRNA‐4	RBH	47.2
		Imd	FBgn0013983	Nlec_unknown_03830‐mRNA‐1	RBH	72.8
		Tak1	FBgn0026323	NA	NA	NA
		IKKBeta	FBgn0024222	Nlec_IKKB‐mRNA‐2	RBH	34.9
		Rel	FBgn0014018	Nlec_NFKB1‐mRNA‐2	RBH	37.2
		Kenny	FBgn0041205	NA	NA	NA
		Fadd	FBgn0038928	Nlec_unknown_05739‐mRNA‐1	RBH	28
		Dredd	FBgn0020381	Nlec_CASP8‐mRNA‐1	RBH	26.1
Gram‐positive	Toll	PGRP‐SA	FBgn0030310	Nlec_unknown_05324‐mRNA‐1	RBH	42.2
		GNBP1	FBgn0040323	Nlec_BGBP1‐mRNA‐4	Nlec_v_Dnel	32.2
		GNBP3	FBgn0040321	Nlec_unknown_04670‐mRNA‐1	RBH	33.2
		Psh	FBgn0030926	Nlec_unknown_06133‐mRNA‐1	Dmel_v_Nlec	32
		modSP	FBgn0051217	Nlec_LFC‐mRNA‐1	RBH	28.1
		SPE	FBgn0039102	Nlec_CA055‐mRNA‐4	RBH	38.5
		Spz	FBgn0003495	Nlec_unknown_05294‐mRNA‐1	RBH	30.2
		Toll	FBgn0262473	Nlec_unknown_01998‐mRNA‐1	RBH	35.2
		Myd88	FBgn0033402	Nlec_MYD88_2‐mRNA‐2	RBH	37.9
		Tub	FBgn0003882	Nlec_unknown_05542‐mRNA‐1	RBH	33.8
		Pll	FBgn0010441	Nlec_ABCB6‐mRNA‐1	Dmel_v_Nlec	64.2
		Dif	FBgn0011274	NA	NA	NA
				Nlec_DORS_2‐mRNA‐1	RBH	65.8
		Dl	FBgn0260632	Nlec_DORS_1‐mRNA‐1	Nlec_v_Dmel	53.9
		Cact	FBgn0000250	Nlec_CACT‐mRNA‐1	RBH	35
NA	JAK/STAT	Upd1	FBgn0004956	NA	NA	NA
		Upd2	FBgn0030904	NA	NA	NA
		Upd3	FBgn0053542	NA	NA	NA
		Dome	FBgn0043903	Nlec_unknown_01653‐mRNA‐1	RBH	26.5
		Hop	FBgn0004864	Nlec_JAK‐mRNA‐1	RBH	42.5
		Stat92E	FBgn0016917	Nlec_STA5B‐mRNA‐8	RBH	49.1
		Ptp61D	FBgn0267487	Nlec_PTP61‐mRNA‐1	RBH	59.5
		RanBP3	FBgn0039110	Nlec_unknown_04013‐mRNA‐1	RBH	35
		Cnot4	FBgn0051716	Nlec_CNOT4‐mRNA‐1	RBH	72
Cellular	Encapsulation/P	Nrg	FBgn0264975	Nlec_unknown_00287‐mRNA‐1	RBH	60.4
		TM9SF4	FBgn0028541	Nlec_TM9S4‐mRNA‐2	RBH	73.4
		ItgaPS4	FBgn0034005	Nlec_ITA3‐mRNA‐1	Dmel_v_Nlec	25.4
		Mys	FBgn0004657	Nlec_unknown_04235‐mRNA‐1	RBH	55.2
		Rac1	FBgn0010333	Nlec_unknown_05689‐mRNA‐1	RBH	92.7
		Rac2	FBgn0014011	NA	NA	NA
		NimC1	FBgn0259896	NA	NA	NA
		Eater	FBgn0243514	Nlec_unknown_05111‐mRNA‐1	RBH	36.9
		Scar	FBgn0041781	Nlec_unknown_05510‐mRNA‐1	RBH	53.5
		Arp2	FBgn0011742	Nlec_unknown_03588‐mRNA‐1	RBH	87.7
		Arp3	FBgn0262716	Nlec_ARP3‐mRNA‐1	RBH	86.6
		Tsr	FBgn0011726	Nlec_CADF_2‐mRNA‐1	RBH	84.4
		Chic	FBgn0000308	Nlec_PROF‐mRNA‐1	RBH	80.2
		Ssh	FBgn0029157	Nlec_SSH1‐mRNA‐1	RBH	75
	Melanization	PPO1	FBgn0283437	NA	NA	NA
		PPO2	FBgn0033367	Nlec_PRPA3_1‐mRNA‐1	RBH	60.1
		PPO3	FBgn0261363	NA	NA	NA

*Note*: Dmel_v_Nlec = best hit for *D. melanogaster* protein against all *N. lecontei* proteins only. Nlec_v_Dmel = best hit for *N. lecontei* protein against all *D. melanogaster* proteins only.

Abbreviation: RBH, reciprocal best hit.

**TABLE A8:** Comparison of olfactory receptor (OR), gustatory receptor (GR), and odorant‐binding protein (OBP) gene families (manually curated gene annotation datasets).

Species	OR intact^a^	OR pseudo	OR partial^b^	GR intact^a^	GR pseudo	GR partial^b^	OBP intact^a^	OBP pseudo	OBP partial^b^	Reference
Sawfly										
*Athalia rosae* (turnip sawfly)	36	–^c^	7	25	–	6				Oeyen et al. (2020)
*Cephus cinctus* (wheat‐stem sawfly)	66	6	0	32	3	0	15	0	0	Robertson et al. (2018)
*Neodiprion lecontei* (redheaded pine sawfly)	52	1	3	41	2	2	13	0	0	This paper
*Orussus abietinus* (wood wasp/parasitoid sawfly)	19	–	13	4	–	3				Oeyen et al. (2020)
Bee										
*Apis cerana* (Asian honey bee)	119	–	–	10	–	–	17	0	0	(OR, GR) Park (OBP) Zhao et al. (2016) (antennal transcriptome)
*Apis florea* (dwarf/red honey bee)	172	7	–	26	2	–	22	2	–	Fouks et al. 20 Updates Karpe et al. (2016)
*Apis dorsata* (giant honey bee)	161	19	–	22	8	–	20	0	–	Fouks et al. (2021)
*Apis mellifera* (European honey bee)	169	8	–	16	10	–	21	0	0	(OR) Robertso (GR) Fouks et (OBP) Forêt and Maleszka (2006)
*Bombus breviceps*	155	–	–	19	–	–				Sun et al. (2021)
*Bombus confusus*	137	–	–	18	–	–				Sun et al. (2021)
*Bombus consobrinus*	153	–	–	17	–	–				Sun et al. (2021)
*Bombus cullumanus*	154	–	–	18	–	–				Sun et al. (2021)
*Bombus difficillimus*	154	–	–	17	–	–				Sun et al. (2021)
*Bombus haemorrhoidalis*	157	–	–	19	–	–				Sun et al. (2021)
*Bombus ignitus*	165	–	–	22	–	–				Sun et al. (2021)
*Bombus opulentus*	162	–	–	19	–	–				Sun et al. (2021)
*Bombus picipes*	156	–	–	18	–	–				Sun et al. (2021)
*Bombus polaris*	152	–	–	20	–	–				Sun et al. (2021)
*Bombus pyrosoma*	153	–	–	18	–	–				Sun et al. (2021)
*Bombus sibiricus*	152	–	–	18	–	–				Sun et al. (2021)
*Bombus skorikovi*	134	–	–	13	–	–				Sun et al. (2021)
*Bombus soroeensis*	165	–	–	18	–	–				Sun et al. (2021)
*Bombus superbus*	133	–	–	17	–	–				Sun et al. (2021)
*Bombus terrestris* (buff‐tailed/large earth bumblebee)	151	5	10	21	3	1	16	–	–	Sadd et al. (2015)
*Bombus turneri*	134	–	–	15	–	–				Sun et al. (2021)
*Bombus waltoni*	137	–	–	17	–	–				Sun et al. (2021)
*Eulaema bombiformis* (orchid bee)	105	2	8	3	0	4	14	0	1	Brand and Ramírez (2017) (antennal transcriptome)
*Euglossa dilemma* (orchid bee)	123	14	42	13	0	0	15	0	0	Brand and Ramírez (2017) (OR table does not match sequence file; reported sequence file here)
*Euglossa flammea* (orchid bee)	105	0	10	3	0	3	14	0	0	Brand and Ramírez (2017) (antennal transcriptome)
*Euglossa imperialis* (orchid bee)	100	0	8	6	0	5	14	0	0	Brand and Ramírez (2017) (antennal transcriptome)
*Euglossa meriana* (orchid bee)	106	1	4	5	0	5	14	0	4	Brand and Ramírez (2017) (antennal transcriptome)
*Euglossa viridissima* (orchid bee)	85	–	3	4	–	1	11	–	0	Brand and Ramírez (2017) (antennal transcriptome)
*Eufriesea mexicana* (orchid bee)	111	12	19	16	0	0	13	1	2	Brand and Ramírez (2017)
*Lasioglossum albipes* (sweat bee)	158	8	18	23	0	4				Zhou et al. (2015)
*Melipona quadrifasciata* (stingless bee)	142	12	42	10	3	3	6	0	7	Brand and Ramírez (2017)
*Dufourea novaeangliae* (solitary bee)	77	11	35							Karpe et al. (2017)
*Habropoda laboriosa* (southeastern blueberry bee)	100	19	51							Karpe et al. (2017)
Ant										
*Acromyrmex echinatior* (leaf‐cutter ant)	375	57	23	116	34	13				Zhou et al. (2015)
*Atta cephalotes* (leaf‐cutter ant)	341	89	65	89	82	14				Zhou et al. (2015)
*Camponotus floridanus* (Florida carpenter ant)	352	–	55	46	–	17	13	–	–	(OR, GR) Zhou (OBP) McKenzie et al. (2014)
*Cardiocondyla obscurior*	232	7	33	34	1	3				Zhou et al. (2015)
*Cerapachys biroi/Ooceraea biroi* (clonal raider ant)	256	141	109	20	–	–	15	–	–	(OR, GR) Oxley (OBP) McKenzie et al. (2014)
*Harpegnathos saltator* (Indian/Jerdon's jumping ant)	347	–	30	17	–	3	13	–	–	(OR, GR) Zhou (OBP) McKenzie et al. (2014)
*Linepithema humile* (Argentine ant)	301	30	6	93	20	4	13	–	–	(OR, GR) Smit (OBP) McKenzie et al. (2014)
*Monomorium pharaonis* (Pharaoh's ant)	240	31	97	159	17	29				Zhou et al. (2015)
*Pogonomyrmex barbatus* (red harvester ant)	274	55	82	58	12	4	16	–	–	(OR, GR) Smit (OBP) McKenzie et al. (2014)
*Solenopsis invicta* (fire ant)	333	66	86	219	60	56	18	–	–	(OR, GR) Zhou (OBP) Gotzek et al. (2011)
Wasp										
*Ampulex compressa* (emerald cockroach wasp)	311	–	–	17	–	–	17	–	–	Obiero et al. (2021)
*Ceratosolen solmsi* (fig wasp)	56	2	1	5	1	0	7	0	–	(OR, GR) Zhou (OBP) Xiao et al. (2013)
*Cerceris arenaria* (sand‐tailed digger wasp)	241	–	–	10	–	–	12	–	–	Obiero et al. (2021)
*Cotesia congregata*	243			54						Gauthier et al. (2021)
*Cotesia flavipes*	202									Gauthier et al. (2021)
*Cotesia rubecula*	296									Gauthier et al. (2021)
*Cotesia sesamiae*	197									Gauthier et al. (2021)
*Cotesia vestalis*	252									Gauthier et al. (2021)
*Diachasma alloeum*	187	14	–	39	1	–	15	0	–	Tvedte et al. (2019)
*Gonatopus flavifemur* (pincer wasp)	43	–	–	10	–	–	8	–	–	Yang et al. (2021)
*Macrocentrus cingulum*	89	–	–	14	–	–	7	–	–	Yin et al. (2018)
*Microplitis demolitor*	203	4	15	79	1	6				Zhou et al. (2015)
*Microplitis mediator*	58	–	2	0	–	2	20	–	–	(OR, GR) Wan (OBP) Peng et al. (2017) (antennal transcriptome)
*Nasonia vitripennis* (jewel wasp)	217	75	9	47	11	0	82	8	0	(OR, GR) Robe (OBP) Vieira et al. (2012)
*Psenulus fuscipennis*	122	–	–	13	–	–	25	–	–	Obiero et al. (2021)

^a^ May include incomplete, pseudogenized annotations.

^b^ The criteria for an intact gene annotation varied across studies. Splice variants were not included.

^c^ Not explicitly declared in publication(s). Value = 0 indicates the genes were searched for but not found. Blank cells indicate the gene family was not studied.

**TABLE A9:** Comparison of select insect antimicrobial peptide gene family sizes (manually curated gene annotation datasets).

Species	Abaecin	Defensin	Tachystatin‐like	Hymenoptaecin	Hisnavicin	Total AMPs^a^	Reference
Sawfly							
*Neodiprion lecontei* (redheaded pine sawfly)	1	0	1	1	8	12	This paper
Bee							
*Apis cerana* (Asian honey bee)	1	2	0	2	0	7	Diao et al. 201 Also 1 apidaecin, 1 apisimin
*Apis mellifera* (European honey bee)	1	2	1	1	0	6	Evans et al. 20 Also 1 apidaecin
*Bombus impatiens* (common eastern bumblebee)	1	1	0	1	0	4	Barribeau et al. Sadd et al. 20 Also 1 apidaecin
*Bombus terrestris* (buff‐tailed/large earthbumblebee)	1	1	0	1	0	4	Barribeau et al. Sadd et al. 20 Also 1 apidaecin
Ant							
*Acromyrmex echinatior* (leaf‐cutter ant)	1	1	2	3	0	8	Gupta et al. (2015)
*Acromyrmex echinatior* (leaf‐cutter ant)	2	1	2	1			Zhang and Zhu (2012)
*Atta cephalotes* (leaf‐cutter ant)	1	1	1	1	0	5	Gupta et al. (2015)
*Atta cephalotes* (leaf‐cutter ant)	2	1	2	1			Zhang and Zhu (2012)
*Camponotus floridanus* (Florida carpenter ant)	0	2	2	1	0	6	Gupta et al. (2015)
*Camponotus floridanus* (Florida carpenter ant)	0	2	3	1			Zhang and Zhu (2012)
*Cerapachys biroi/Ooceraea biroi* (clonal raider ant)	1	1	3	1	0	6	Gupta et al. (2015)
*Harpegnathos saltator*(Indian/Jerdon's jumping ant)	1	2	2	2	0	8	Gupta et al. (2015)
*Harpegnathos saltator* (Indian/Jerdon's jumping ant)	1	2	3	2			Zhang and Zhu (2012)
*Linepithema humile* (Argentine ant)	0	1	3	1	0	6	Gupta et al. (2015)
*Linepithema humile* (Argentine ant)	0	1	2	1			Zhang and Zhu (2012)
*Pogonomyrmex barbatus* (red harvester ant)	1	5	2	1	0	10	Gupta et al. (2015)
*Pogonomyrmex barbatus* (red harvester ant)	2	6	5	1			Zhang and Zhu (2012)
*Solenopsis invicta* (fire ant)	0	2	3	1	0	7	Gupta et al. (2015)
*Solenopsis invicta* (fire ant)	2	6	9	1			Zhang and Zhu (2012)
Wasp							
*Ceratosolen solmsi* (fig wasp)	1	0	0	0	1	8	Xiao et al. (2013)
*Cotesia congregata*				1		1	Gauthier et al. (2021)
*Nasonia vitripennis* (jewel wasp)	3	6	3	2	5	44	Tian et al. (2010)
*Cotesia vestalis*	–^b^	10	–	1	–	15	Shi et al. (2019) Also 3 ixodidin, 1 enhancer of rudimentary homolog
*Diadromus collaris*	–	8	–	1	–	14	Shi et al. (2019) Also 1 ixodidin, 1 crustin, 2 secretory leukocyte protease inhibitor, 1 enhancer of rudimentary homolog

^a^ The criteria for an intact gene annotation varied across studies. Splice variants were not included.

^b^ Not explicitly declared in publication(s). Value = 0 indicates the genes were searched for but not found. Blank cells indicate the gene family was not studied.
